# The *gac* system integrates physical and chemical cues to promote plant root attachment

**DOI:** 10.1128/aem.00778-26

**Published:** 2026-06-26

**Authors:** Guy Sobol, David M. Hershey

**Affiliations:** 1 Department of Bacteriology, University of Wisconsin-Madison 205263, Madison, Wisconsin, USA; The University of Tennessee Knoxville, Knoxville, Tennessee, USA

**Keywords:** rhizosphere, plant roots, biofilm, *gac *system signaling

## Abstract

Plants host complex communities of microbes that are attached to root surfaces. While many studies have sampled mutant populations after prolonged incubation on roots to identify bacterial genes that enable long-term colonization, the molecular mechanisms governing the early stages of root attachment remain less understood. Here, we developed an *in vitro* root culture system that enables controlled and scalable investigation of bacterial attachment to root tissue. We used this platform to perform a genome-wide screen for root attachment determinants in the plant-associated bacterium *Pseudomonas protegens* Pf-5. Our results reveal that the *gacSA* two-component system functions as a sensory integration hub for coordinating early root attachment. Mutations that disrupt *gacS* or *gacA* cause severe root attachment defects despite having no effect on abiotic surface attachment in standard biofilm assays. Mutation of flagellar assembly genes enhances root attachment by mimicking surface contact and activating the *gac* system. In parallel, chemical cues released by roots stimulate surface attachment in a *gac-*dependent manner. By integrating these signals, the *gac* system activates cyclic-di-GMP-mediated attachment programs that drive the transition from planktonic to sessile behavior required for root association. We build on this model to show that manipulating flagellar surface-sensing sustains the attachment of Pf-5 in the presence of a synthetic bacterial community, suggesting a strategy to improve the attachment of beneficial microbes on crops. These findings establish a mechanistic framework linking surface sensing, global regulation, and root attachment in a beneficial rhizobacterium.

**IMPORTANCE:**

Plant roots are covered with diverse microbes that strongly influence plant health. Growing in association with roots has many benefits, but how bacteria attach to root tissue remains poorly understood. We developed a system to study how the plant-associated bacterium *Pseudomonas protegens* Pf-5 attaches to roots. We found that physical contact with the root surface and chemical cues released by roots both enhance attachment to roots. A sensory system called *gacSA* is responsible for integrating physical and chemical cues to activate a root attachment program. Variant bacteria that prematurely activate the *gac* system attach more effectively even in the presence of other bacteria on roots, suggesting that the root attachment pathway we characterized could serve as a strategy to use beneficial bacteria in agriculture.

## INTRODUCTION

Surface-associated communities called biofilms represent the predominant lifestyle for most bacteria (1, 2). Cells in biofilms are encased in a self-produced extracellular matrix that provides resistance to stresses such as shear flow, unfavorable temperatures, antimicrobial substances, and nutrient limitation (3). While biofilms are found in nearly all environments on Earth, their association with living tissues has particularly significant effects on humans. Biofilms within human tissues are thought to be the source of chronic infections, and tolerance to antimicrobial treatments makes them difficult to treat in clinical settings (4). In agricultural systems, root colonization by beneficial bacteria, often through biofilm formation, can influence crop productivity (5). Despite the profound importance of host-associated biofilms, mechanistic analyses of biofilm formation have historically been dominated by studies on abiotic surfaces such as plastic, glass, and agar. Knowledge of host-associated biofilms is often inferred from these inanimate models (6). Some of the earliest studies of host-associated biofilms were conducted in the oral cavity, where attachment to tooth enamel and epithelial surfaces provided foundational insights into surface colonization (7, 8). More recently, substantive efforts to study bacterial colonization of biotic substrates, including plant tissues and animal-associated surfaces such as the intestine, spleen, and others, have begun to reveal differences in host-associated biofilm formation, but continued work is needed to accelerate the development of strategies for managing biofilms in host-associated settings (9).

**Fig 1:**
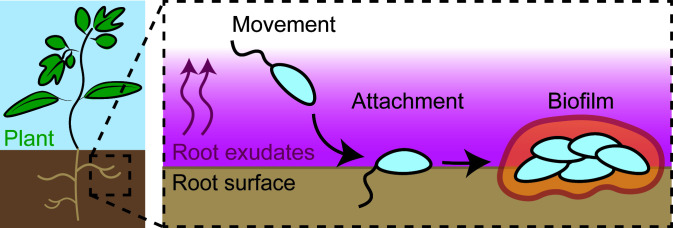
Bacterial attachment to root surfaces. Roots release a mixture of exudates into the surrounding soil, generating a concentration gradient that is highest near the root surface (illustrated as a pink gradient). Rhizosphere bacteria actively move toward roots based on the exudate gradient, attach to the root surface via secreted adhesins, and subsequently form a biofilm.

Plant health is strongly influenced by complex biofilms in the rhizosphere, a narrow zone of soil surrounding root tissues (10–12). Plants interact with microbes in the rhizosphere by secreting sugars, amino acids, organic acids, and secondary metabolites that are collectively referred to as exudates (13, 14). Exudates create chemical gradients that attract bacteria toward the root surface (Fig. 1). They also promote bacterial aggregation and provide nutrients that make the root surface a favorable niche. Plants, in turn, rely on microbes in the rhizosphere for nutrient acquisition, suppression of pathogenic organisms, and tolerance to stressors such as drought. This mutualistic relationship requires microbes to physically attach to the root surface.

Efforts to identify the genetic determinants of root attachment have largely relied on inoculating mutant libraries onto plants growing in substrates such as soil, vermiculite, sand, or synthetic plant media. Sampling the root-associated population using transposon sequencing (Tn-Seq) has consistently identified genes involved in amino acid biosynthesis, carbon utilization, nitrogen assimilation, iron acquisition, suppression of plant defenses, and competition with other microbes (15–24). Such studies have been instrumental in defining metabolic pathways required for persistence in the rhizosphere. However, the prolonged incubations in these studies emphasize sustained colonization rather than the earliest stage of root attachment. A more thorough understanding of how bacteria attach to root tissue is needed to understand biofilm formation in the rhizosphere.

Members of the *Pseudomonas fluorescens* species complex are frequent residents of the rhizosphere (25, 26). These bacteria often promote plant health by enhancing nutrient uptake, producing growth-promoting compounds, and antagonizing pathogens. There is extensive knowledge of the mechanisms that govern biofilm formation within the *Pseudomonas* genus, particularly in the opportunistic human pathogen *Pseudomonas aeruginosa* (27). *Pseudomonads* coordinate biofilm formation through a regulatory paradigm centered on the second messenger cyclic-di-GMP (c-di-GMP). While the details of each signaling cascade vary among individual strains, environmental cues trigger elevated c-di-GMP levels, which, in turn, bind effector proteins that promote biofilm-associated behaviors, such as reduced motility, biofilm matrix production, and expression of adhesins. A well-characterized output of c-di-GMP signaling in *P. fluorescens* is the Lap system, which controls a large cell-surface adhesin, LapA. Elevated intracellular c-di-GMP concentrations promote the accumulation of LapA at the cell surface, where it mediates attachment and supports biofilm formation. Reduced c-di-GMP levels result in LapA cleavage and release, leading to detachment and dispersal (28).

In this study, we develop an *in vitro* root tissue culture system to investigate bacterial root attachment. This approach overcomes practical limitations of studying root attachment in whole plants and avoids abiotic adhesion assays that do not capture interactions with living host tissue. We use *Pseudomonas protegens* Pf-5, a soil bacterium that associates with the roots of various crops (29–32), to perform a genome-wide screen for root attachment determinants. Our results demonstrate that the *gacSA* two-component system acts as a key integrator of signals to coordinate root attachment. Both flagellum-mediated surface sensing and chemical cues released by roots activate *gac* signaling to promote c-di-GMP-dependent attachment. Furthermore, we show that manipulating flagellar surface sensing enhances the attachment of Pf-5 in the presence of a synthetic bacterial community, suggesting a potential strategy for improving the performance of beneficial bacteria in agricultural applications. Our results shed new light on how host tissues influence the earliest stages of biofilm formation.

## RESULTS

### Genome-wide screen identifies *P. protegens* Pf-5 genetic determinants for early root attachment

**Fig 2:**
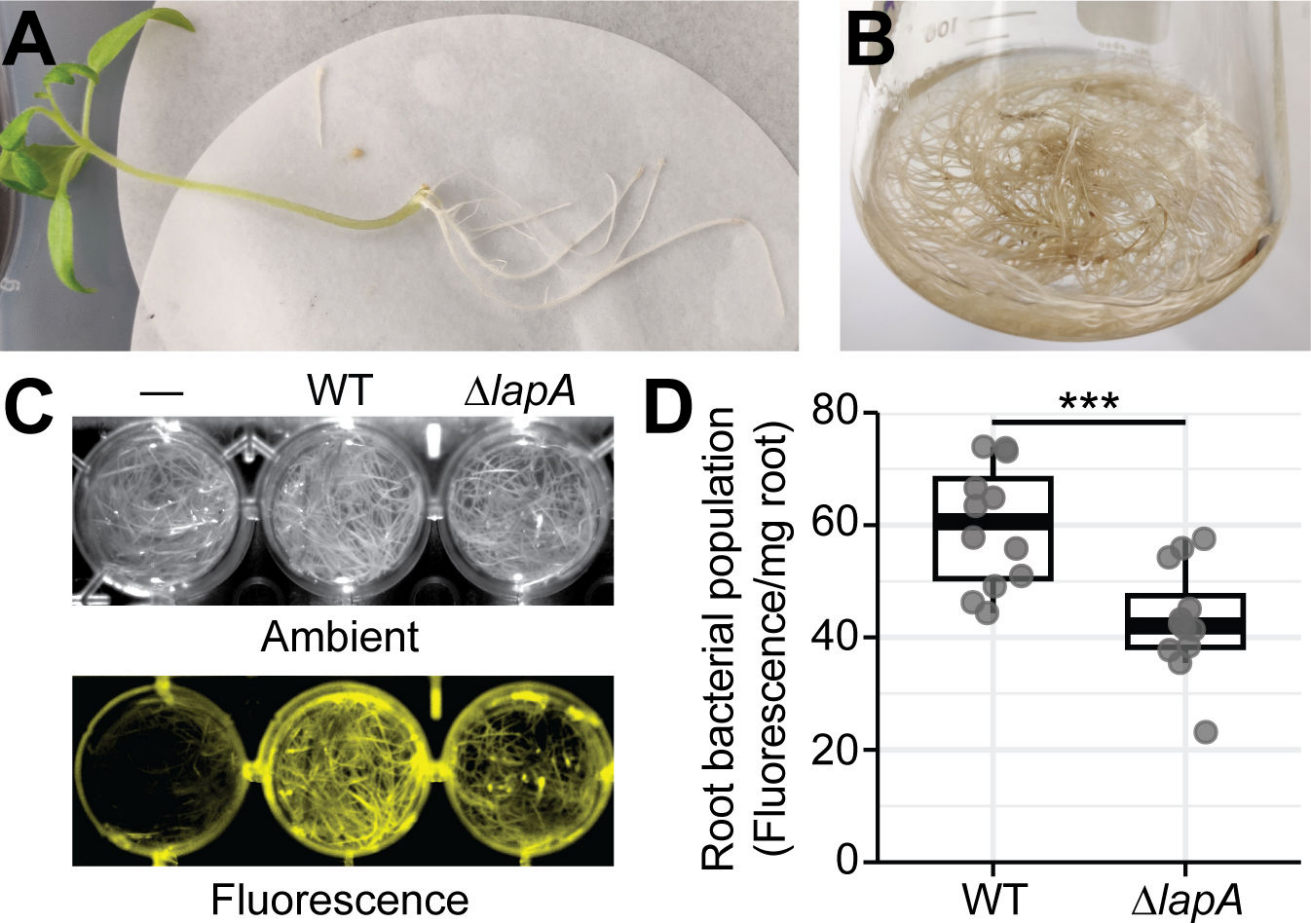
Establishing an *in vitro* root tissue culture system to study root colonization. (**A**) Seedlings grown under sterile tissue culture conditions were inoculated with *Agrobacterium* at an incision at the base of the hypocotyl to generate hairy roots. (**B**) Excised root tissue was transferred to liquid medium and propagated in shaking culture, resulting in root biomass suitable for downstream colonization assays. (C and D) Root tissue placed in plate wells was inoculated with a bacterial suspension of mVenus-tagged Pf-5 wild-type or Δ*lapA*. Root colonization was evaluated by measuring fluorescence from roots (**C**) or quantified by recovering the root-attached bacterial fraction and measuring its emitted fluorescence (**D**). Bold lines within each box represent median values. Top and bottom sides of the boxes represent the third and first quartiles of the value distribution, respectively. Lines extending from the boxes denote the extreme values within 1.5 times the interquartile range. Statistical significance was determined by a pairwise *t*-test. Asterisks indicate *P* < 0.001. Data are pooled from three independent experiments.

We developed an *in vitro* tissue culture system for propagating tomato roots in liquid medium (Fig. 2A and B). This platform allows large quantities of root tissue to be cultivated independently of whole plants under highly controlled conditions. The resulting roots are anatomically indistinguishable from primary and adventitious roots (33) and maintain exudation patterns similar to those of the parent plant roots (34). The abundance of root tissue increases the sensitivity of the assay, enabling attachment to be quantified at early time points when bacterial colonization is lower. In addition, both the roots and the planktonic bacterial population can be readily recovered: roots are directly accessible and do not require excavation from soil and washing, and planktonic cells can be sampled directly from the culture medium. We then engineered a Pf-5 strain expressing mVenus and used it to carefully optimize conditions for robust colonization of root tissue. Pf-5 did not grow detectably in standard root culture medium, but adding carbon sources commonly detected in root exudates allowed it to grow (Fig. S1A through C) (35). We therefore included succinate in the root growth media to maintain cells in a metabolically active state during colonization assays. We validated our system by comparing root association of fluorescently tagged wild-type Pf-5 to a mutant lacking the large surface adhesin LapA (28). Bacterial abundance on roots was measured by imaging the total emitted fluorescence from roots in plate wells (Fig. 2C), measuring the fluorescence emitted by cells recovered from roots, or directly quantifying viable cell counts (Fig. 2D and Fig. S1D). Both strains attached to root tissue, but roots inoculated with the Δ*lapA* mutant emitted reduced levels of fluorescence and carried fewer CFUs compared with wild-type Pf-5, consistent with a deficiency in root attachment.

**Fig 3:**
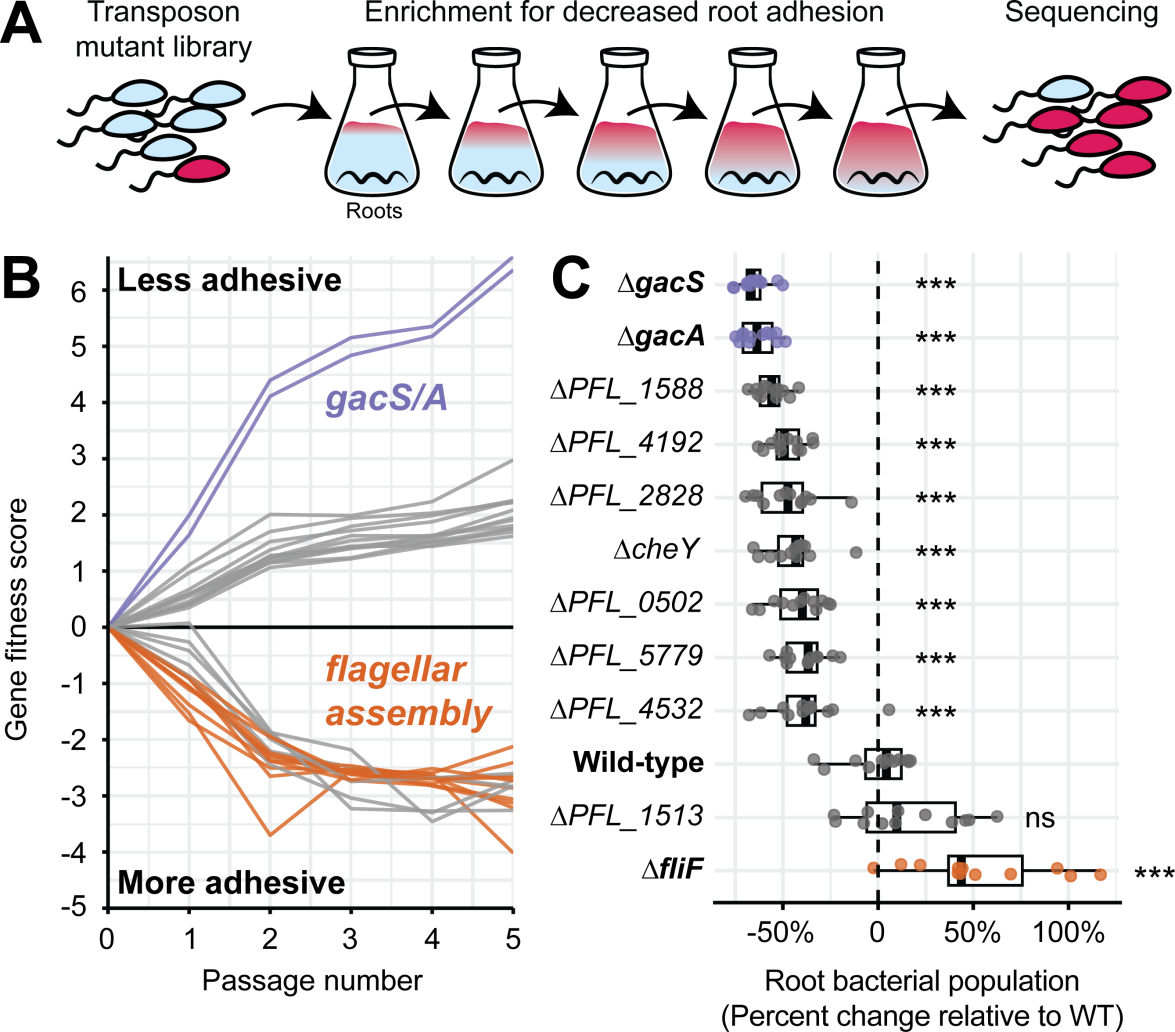
A genome-wide screen identifies genetic determinants for root attachment. (**A**) A pooled transposon mutant library carrying mutants impaired in root association (red) was inoculated into flasks containing hairy roots. After incubation, the unattached (planktonic) fraction was collected and serially passaged into fresh root-containing flasks for five rounds. The resulting population is enriched for adhesion-defective mutants and sequenced. (**B**) Fitness trajectories for the 15 most hypo- and 15 most hyper-adhesive mutants were identified in the root passaging Tn-Seq screen. Lines connect fitness scores for each gene across passages 0–5. Positive fitness scores indicate mutants that increased in abundance in the unattached (media) fraction, consistent with reduced root adhesion, whereas negative scores reflect depletion from the media and therefore enhanced adhesion to roots. Genes highlighted in purple represent the *gacS* and *gacA* genes, orange denotes flagellar assembly-associated genes, and gray indicates genes outside these categories. (**C**) Root colonization phenotypes for in-frame deletion mutants of genes identified in the screen. Root colonization was quantified by recovering the root-attached fraction of cells and measuring its emitted fluorescence. Bold lines within each box represent median values. Top and bottom sides of the boxes represent the third and first quartiles of the value distribution, respectively. Lines extending from the boxes denote the extreme values within 1.5 times the interquartile range. Statistical significance was determined by a pairwise *t*-test between wild-type and each strain. Asterisks indicate *P* < 0.001; “ns,” no significant difference. Data are pooled from three independent experiments.

We used the *in vitro* colonization system to perform a genome-wide screen for Pf-5 genes involved in attachment to roots. A barcoded transposon mutant library was inoculated into flasks containing root tissue, and the mutant pool was allowed to attach to roots for 24 h, an early time point compared to other root colonization studies that typically assess colonization after several days, 1 week, or longer (9, 36). We predicted that mutants that retained the ability to associate with root tissue would partition out of the medium as they attached to roots, enriching for attachment-deficient mutants in the liquid medium. We amplified these selections by inoculating the unattached population into fresh root tissue for four additional passages (Fig. 3A). Barcode abundances from the initial mutant population and from the unattached fraction after each root passage were used to calculate fitness scores for each gene across the passages (Fig. 3B and Table S1).

Genes with the strongest effects on root attachment are shown in Tables 1 and 2. Mutations causing the strongest defects in root attachment mapped to *gacS* and *gacA,* which encode a histidine kinase-response regulator pair that sits atop a large regulatory hierarchy. Mutations affecting transcription factors (*PFL_2828*, *PFL_0502*, and *PFL_1588*), uncharacterized transporters (*PFL_4192* and *PFL_1740*), the *pxpABC* operon encoding the allophanate hydrolase enzyme (*PFL_1513-1515*) (37), as well as genes involved in c-di-GMP (*PFL_5779* and *PFL_4532*), lipid (*PFL_5687*), and carbon (*PFL_6157*) metabolism also caused root attachment deficiencies. Mutations that enhanced root attachment were concentrated in genes required for the function of the flagellum, including assembly regulators, structural components, and chemotaxis systems (38). In-frame deletion mutants Δ*gacS* and Δ*gacA* recapitulated the strong attachment defect detected in the Tn-Seq, where they were the most depleted mutants, and similarly exhibited the strongest attachment defects in the targeted root attachment assays, with root attachment reduced by ~60% relative to wild type (Fig. 3C). The Δ*fliF* mutant, representing flagellar structural mutants and lacking a flagellum, attached ~30% more effectively than wild type. Deletion mutants of *PFL_1588*, *PFL_4192*, *PFL_2828*, *PFL_0502*, *PFL_5779*, and *PFL_4532* were hypoadhesive, consistent with the Tn-Seq results. The Δ*cheY* mutant was hypoadhesive despite appearing hyperadhesive in the Tn-Seq results. Δ*PFL_1513* did not differ significantly from wild type, although it was hypoadhesive in the Tn-Seq results. All the deletion mutants displayed comparable growth kinetics to wild type (Fig. S2A).

**TABLE 1:** Top 15 protein-encoding hits enriched in the non-root-associated fraction (reduced attachment)

Gene	Locus ID	Gene annotation	Area under curve
*gacS*	PFL_4451	Sensor protein GacS	19.20
*gacA*	PFL_3563	Response regulator GacA	18.13
	PFL_2828	LysR family transcriptional regulator	8.27
	PFL_4192	ABC transporter substrate-binding protein	7.27
*adrA*	PFL_4532	Diguanylate cyclase	6.58
	PFL_0502	DeoR family transcriptional regulator	6.58
*pxpB*	PFL_1514	Allophanate hydrolase subunit 1	5.84
	PFL_1588	AraC family transcriptional regulator	5.61
*pxpA*	PFL_1513	LamB/YcsF family protein	5.48
*pxpC*	PFL_1515	Allophanate hydrolase subunit 2	5.35
	PFL_5687	Acyl-CoA dehydrogenase	5.13
	PFL_1740	TonB-dependent outer membrane receptor	5.13
	PFL_5779	response regulator/sensory box/GGDEF domain/EAL domain-containing protein	5.07
*pycA*	PFL_6157	Pyruvate carboxylase subunit B	4.79
	PFL_2692	Hypothetical protein	4.61

**TABLE 2:** Top 15 protein-encoding hits depleted from the non-root-associated fraction (enhanced attachment)

Gene	Locus ID	Gene annotation	Area under curve
*fleE*	PFL_1637	Flagellar hook-basal body complex protein FliE	11.63
*cheA*	PFL_1670	Chemotaxis protein CheA	10.58
	PFL_2045	Amino acid aminotransferase	9.93
*fliF*	PFL_1638	Flagellar basal-body MS-ring/collar protein FliF	9.81
*flgF*	PFL_1613	Flagellar basal-body rod protein FlgF	9.74
*fliI*	PFL_1641	Flagellar protein export ATPase FliI	9.64
*fliG*	PFL_1639	Flagellar motor switch protein FliG	9.53
*fliA*	PFL_1667	RNA polymerase sigma factor FliA	9.43
*flgE*	PFL_4477	Flagellar hook protein FlgE	9.43
*fliK*	PFL_1646	Flagellar hook-length control protein FliK	9.42
*flgG*	PFL_1614	Flagellar basal-body rod protein FlgG	9.39
*flgC*	PFL_4479	Flagellar basal body rod protein FlgC	9.16
*cheY*	PFL_1668	Chemotaxis response regulator CheY	9.13
*fleR*	PFL_1636	Sigma-54 dependent transcriptional regulator	9.11
	PFL_1752	HAMP domain-containing sensor histidine kinase	8.85

### Flagellar-dependent surface-sensing activates the *gac* system

**Fig 4:**
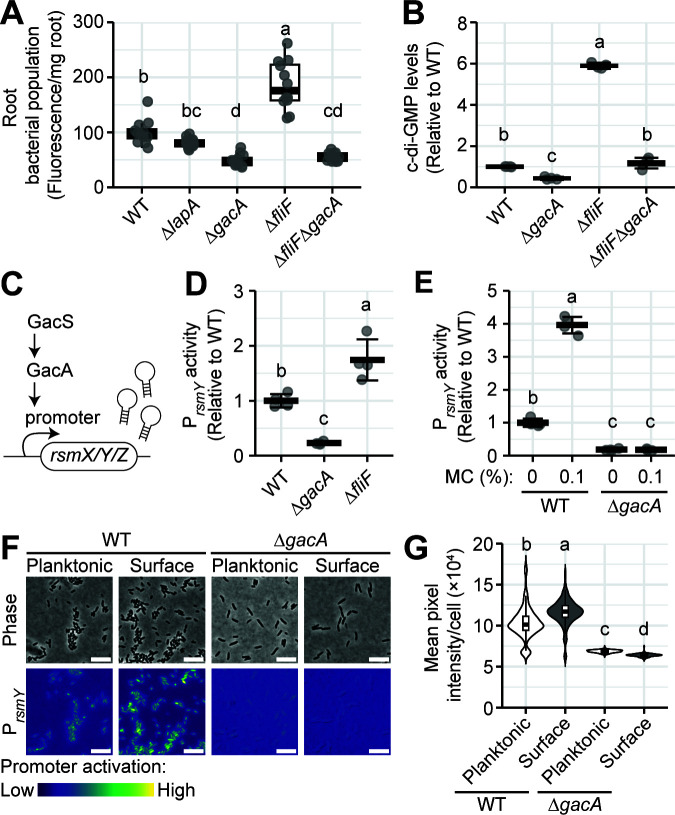
Flagellar-dependent surface sensing activates the *gac* system. (**A**) Root colonization efficiencies of key mutants. Top and bottom sides of the boxes represent the third and first quartiles of the value distribution, respectively. Lines extending from the boxes denote the extreme values within 1.5 times the interquartile range. Data are pooled from three independent replicates. (**B**) Intracellular c-di-GMP levels of strains from panel A. Reporter values were normalized to wild type. (**C**) Overview of the *gac* signaling system in *Pseudomonads*. GacS activates the response regulator GacA, which in turn directly induces the expression of the small RNAs *rsmX*, *rsmY*, and *rsmZ*. In D-G, the effect of surface sensing on *gac* activity was evaluated via P*_rsmY_* expression by disrupting flagellar assembly (Δ*fliF*) (**D**), increasing medium viscosity with methyl cellulose (MC) (**E**), or comparing planktonic and surface-attached cells (**F and G**). In F, scale bars represent 5 μm. In B, D, and E, a representative replicate of three individual experiments is shown. Data are means ± SD. In all panels, letters represent statistical significance determined by one-way ANOVA and Tukey’s post hoc test (*P* < 0.05).

We sought to understand two groups of genes with opposing effects on root colonization. *gacS* and *gacA* mutants have severe root attachment defects (Fig. 3). Activation of the *gacSA* two-component system induces the expression of small RNAs called *rsm,* which go on to activate the genes involved in biofilm formation, secondary metabolite production, and exopolysaccharide synthesis (39). Mutating flagellar genes in Pf-5 strongly enhances root attachment (Fig. 3). The flagellum plays numerous roles throughout the process of biofilm formation, including facilitating productive contact with surfaces and recognizing physical cues for contact with surfaces (38, 40). We confirmed that the root colonization phenotypes of Δ*gacS*, Δ*gacA*, and Δ*fliF* were complemented by ectopic expression of arabinose-inducible forms of each mutated gene (Fig. S2B). We then used epistasis analysis to determine whether the *gac* and flagellar genes contributed to a unified pathway controlling root attachment. A Δ*fliF*Δ*gacA* double mutant displayed a root colonization defect comparable to the ∆*gacA* single mutant, indicating that the *gac* system is required for enhanced root attachment in flagellar mutants (Fig. 4A and Fig. S3). To determine whether differences in root attachment correlate with altered c-di-GMP levels, which are known to promote bacterial adhesion during responses to surface contact, we quantified c-di-GMP levels using a fluorescent reporter (41). The Δ*fliF* mutant showed elevated c-di-GMP, and the Δ*gacA* mutant exhibited lower c-di-GMP concentrations than wild-type (Fig. 4B). The Δ*fliF*Δ*gacA* double mutant displayed c-di-GMP levels matching the wild-type phenotype and confirming that the hyperadhesive, high-c-di-GMP state of Δ*fliF* depends on *gacA*.

Mutations that disrupt flagellar assembly enhance biofilm formation in several bacteria by activating surface-sensing pathways (38, 40). We predicted that the Δ*fliF* mutation enhances adhesion by mimicking surface contact, which in turn activates the *gac* system. We monitored transcriptional reporters for the three small RNAs that are directly activated by GacA ~P in Pf-5 (*rsmX*, *rsmY*, and *rsmZ*) under conditions designed to induce surface sensing (Fig. 4C) (42, 43). Surface sensing was stimulated by increasing medium viscosity with methyl cellulose (44), by disrupting flagellar assembly using the Δ*fliF* mutation, or by incubating cells on an agar surface. *gac* activity was elevated across all conditions where surface sensing was stimulated (Fig. 3D through G). Similar trends were observed for all three *rsm* reporters (Fig. S4). Together, these findings support a model in which physical contact with surfaces influences *gac* system activity through the flagellum.

### The *gac* system integrates physical and chemical cues to control surface attachment

**Fig 5:**
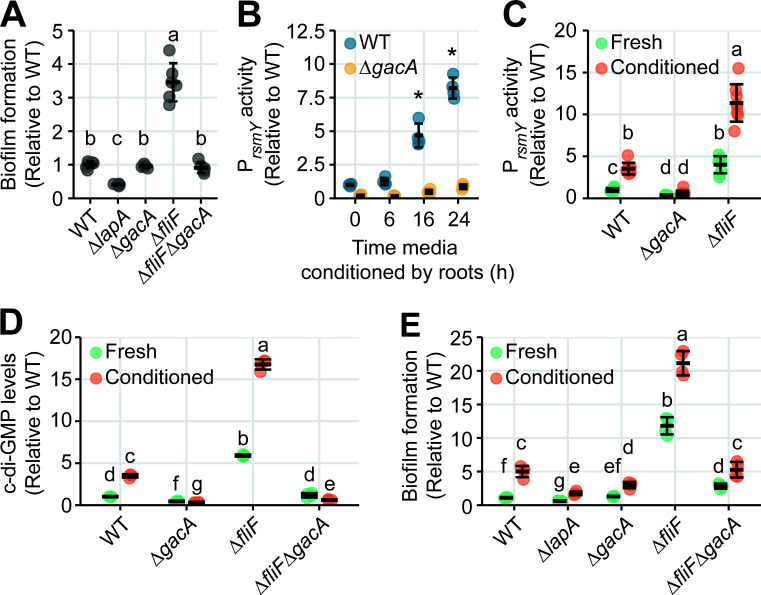
The *gac* system integrates physical and chemical cues to control surface attachment. (**A**) Crystal violet (CV)-based adhesion assay evaluating attachment to an abiotic surface. (**B**) *gac* system activity was quantified using a fluorescent reporter for *rsmY,* evaluating the effect of conditioning time of media by roots on *gac* system activity. (**C–E**) Integration of chemical and mechanical inputs by the *gac* system. Indicated strains were analyzed under conditions providing mechanical input alone (Δ*fliF*), chemical input alone (root-conditioned media), or both inputs combined. (**C**) *gac* system activity quantified via the *rsmY* fluorescent reporter. (**D**) Intracellular c-di-GMP levels. (**E**) CV-based adhesion assay. *gac* system activity levels, c-di-GMP levels, and adhesion were normalized to wild-type in unconditioned (Fresh) media. A representative replicate of three independent experiments is shown. Data represent means ± SD. In all panels, different letters indicate statistically significant differences determined by one-way ANOVA with Tukey’s post hoc test (*P* < 0.05). In panel B, asterisks indicate statistically significant differences determined by pairwise *t*-tests comparing each time point to the preceding time point within the same strain.

To evaluate how the attachment phenotypes we measured on root tissue compared to abiotic surface attachment, we quantified biofilm formation in microtiter plates using a crystal violet (CV) staining assay that stains cells and the extracellular polysaccharides they produce. We observed a different attachment pattern on an abiotic surface than we measured on root tissue. The ∆*fliF* mutant remained hyperadhesive on abiotic surfaces, and a Δ*fliF*Δ*gacA* double mutant still lost the enhanced adhesion of Δ*fliF* alone (Fig. 5A). However, the ∆*gacA* single mutant and the Δ*fliF*Δ*gacA* double mutant showed identical levels of CV staining to the wild-type strain. These results indicate that the *gac* system plays a stronger role in attachment to tissue than to abiotic surfaces.

We predicted that differences in colonization efficiencies of *gac* mutants on root tissues compared to abiotic surfaces could be explained by the presence of exudates that accumulate in tissue culture medium (45, 46). We measured *gac* reporter activity in media that had been conditioned by roots for 6, 16, and 24 h. Activation of all three *gac* reporters increased with longer root conditioning times. No activation was observed in the Δ*gacA* mutant (Fig. 5B and Fig. S5A and C). These results demonstrate that root-secreted molecules stimulate *gac* signaling.

We next tested whether the chemical signals from root exudates and physical signals from the flagellum activate the *gac* system in an additive manner or through a saturating logic in which each cue alone is sufficient to fully activate the response. To distinguish these possibilities, we examined *gac* activity, c-di-GMP levels, and biofilm formation under conditions with surface sensing activated or chemical cues alone, or both inputs together. In an additive model, each input would independently increase reporter output, and combined exposure to both signals would produce a further increase beyond either condition alone. In contrast, in a saturating model, either signal alone would maximally activate the pathway, such that simultaneous exposure to both cues would not further increase reporter output beyond that observed with each individual signal. When we combined the two signals using root-conditioned media and a Δ*fliF* mutant, we observed higher levels of *gac* reporter activation, higher c-di-GMP levels, and increased biofilm formation, compared to conditions with either input alone (Fig. 5C through E and Fig. S5B and D). This enhancement supports the possibility that the *gac* system integrates physical and chemical signals additively to promote root colonization.

### Activation of flagellar surface sensing sustains root attachment in mixed bacterial communities

**Fig 6:**
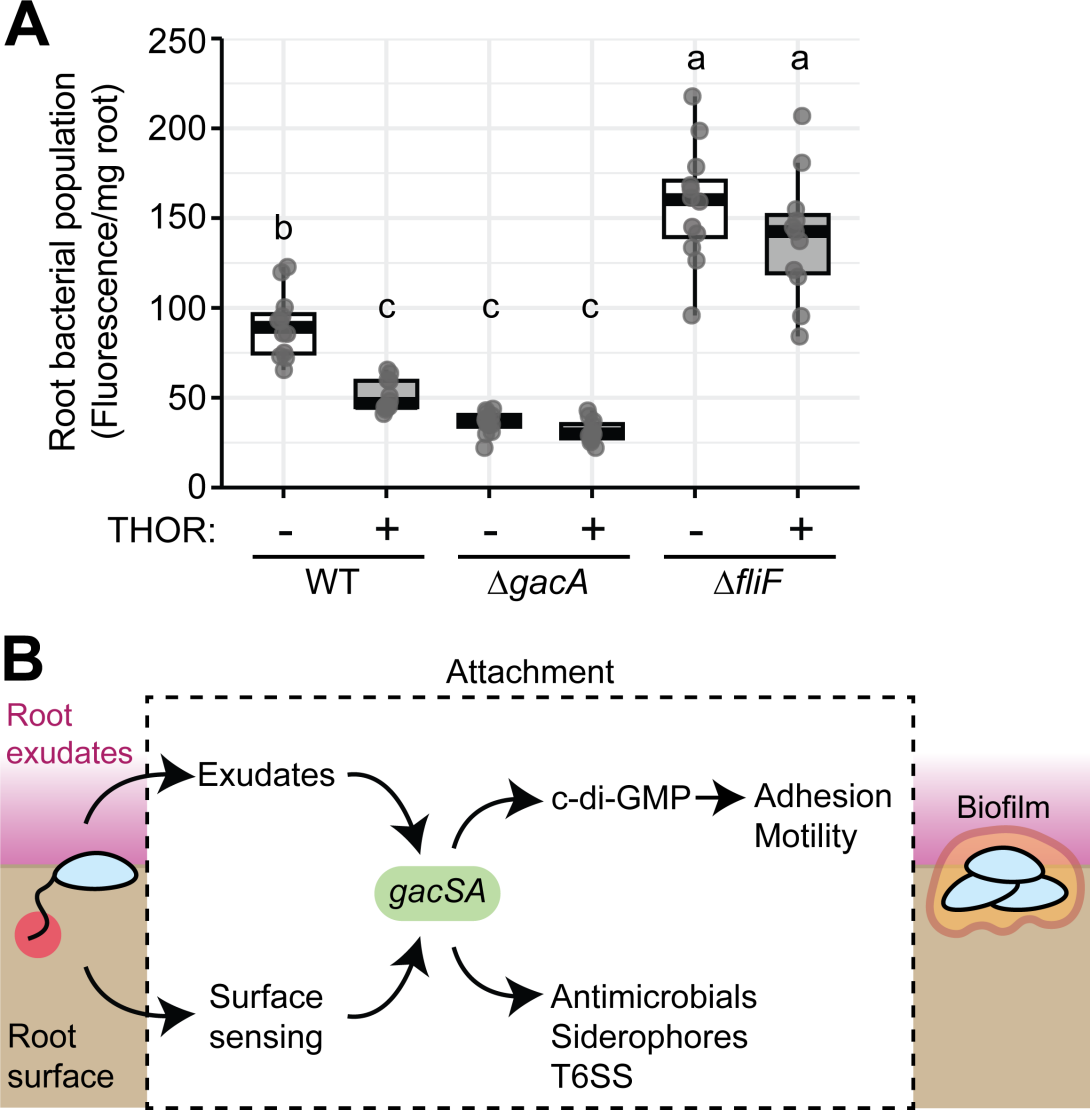
Activating flagellar surface sensing enhances competitive root colonization. (**A**) Root colonization phenotypes in the presence of a synthetic bacterial community. Root colonization was quantified by recovering the root-attached fraction of cells and measuring its emitted fluorescence. Bold lines within each box represent median values. Top and bottom sides of the boxes represent the third and first quartiles of the value distribution, respectively. Lines extending from the boxes denote the extreme values within 1.5 times the interquartile range. Data are pooled from three independent replicates. (**B**) The *gac* system additively integrates physical and chemical cues to coordinately enhance adhesion and reduce motility through c-di-GMP, while simultaneously activating competitive traits, together enabling effective root attachment.

Rhizosphere colonization efficiency is thought to be driven by competitive interactions among soil organisms. We predicted that activating surface responses and *gac* signaling using the Δ*fliF* mutation would provide Pf-5 an advantage during the attachment phase of root colonization in the presence of other bacteria. To test this, we performed a short-term root attachment assay in the presence of THOR, a synthetic bacterial community composed of three members isolated from the soybean rhizosphere (47). Co-inoculation with THOR reduced the attachment efficiency of wild-type Pf-5 by ~40%, while the Δ*gacA* mutant remained at low abundance (Fig. 6A). In contrast, the Δ*fliF* mutant maintained elevated attachment efficiency in the presence of THOR. These results show that enhanced activation of attachment programs in the Δ*fliF* mutant is sufficient to retain root attachment efficiency in the presence of other bacteria in the root environment.

## DISCUSSION

Previous studies have used whole plants to identify bacterial genes that promote persistent colonization of roots (9). These efforts have yielded foundational insights into plant-microbe interactions in the rhizosphere, but the crucial stage of early root attachment has remained less characterized. In this study, we developed an *in vitro* root tissue culture system to specifically target the early stages of bacterial root attachment. The higher throughput, ease of sampling cells, and precise experimental control allowed us to identify biologically relevant phenotypes (Fig. S3A) not apparent in previous studies (9). For example, only a subset of the attachment determinants we found were identified when Pf-5 mutants were inoculated onto wheat and cotton seeds, grown in a sand/perlite mix, and recovered after 1 week (22). More broadly, the results of many long-duration root colonization Tn-Seq studies are dominated by genes involved in metabolism, transport, cell wall biogenesis, and stress adaptation rather than classical biofilm factors (9, 15, 17, 19, 21–24, 26, 48). These contrasts highlight the complex temporal progression of root attachment. Individual genes likely play stage-dependent roles, and early advantages may later become neutral or even detrimental as colonization progresses. Targeting the early attachment phase specifically led to the identification of new signaling connections that influence attachment to roots.

The *gacSA* two-component signaling system emerged as a crucial determinant of root attachment in our study. While the *gac* system is known to influence a large set of behaviors in *Pseudomonads,* there are conflicting reports about its role in biofilm formation and tissue colonization. *gac* mutants generally show reduced biofilm formation on abiotic surfaces in crystal violet assays (49–57). However, other studies indicate that abiotic surface biofilm formation phenotypes of *gac* mutants are inconsistent and may depend on specific culture conditions (58–60). In root colonization assays with whole plants, the consequences of *gac* disruption are strongly shaped by competition. *gac* mutants often do not show impaired root colonization and may even outperform wild type in competition (61, 62), reflecting a public goods tradeoff (56, 63). In contrast, *gac* mutants show a competitive disadvantage in natural soil or mixed-community environments (64–66). While previous Tn-Seq colonization studies that selected for competitive growth identified *gac*, it was not among the strongest contributors to root colonization (18, 22). In our study, *gacS* and *gacA* mutants displayed the strongest colonization defects of any mutants identified. Nevertheless, these mutants were not completely defective in attachment, indicating that additional mechanisms also contribute to root colonization. We propose that *gac* activation is crucial for promoting tissue adhesion specifically during the earliest stages of root contact.

Our study also illuminates a novel function for flagella in the root colonization sequence. Flagella have long been recognized as surface colonization factors, but previous root attachment studies have identified a range of phenotypes for flagellar mutants. Root attachment defects, neutral colonization phenotypes, and hyper-colonization of roots have all been reported (17, 48, 64, 67, 68). Variations likely arise because specific experimental methods inadvertently emphasize different stages of root colonization, but previous studies have mostly interpreted flagellar phenotypes through the lens of motility. Colonization defects are attributed to an inability to migrate efficiently to the root surface, and hyper-colonization is assumed to arise when cells become “trapped” on the root because they cannot swim away. Our root colonization screen revealed an additional role for the flagellum beyond motility. Because the assay was performed under agitation, bacterial cells were continuously brought into contact with the root surface, minimizing the requirement for motility to reach the root. Under these conditions, flagellar mutants displayed a hyperadhesive phenotype, indicating that the flagellum influences attachment after root contact. Specifically, our results support a role for the flagellum as a surface sensor. The enhanced adhesion and elevated c-di-GMP levels of the Δ*fliF* mutant are consistent with studies showing that disrupting flagellar assembly mimics surface contact (69, 70). Moreover, we could recapitulate the effects of the ∆*fliF* mutant in wild-type cells by physically obstructing the flagellum (Fig. 4D through G and Fig. S4). Thus, our results provide evidence that surface-sensing mechanisms identified for abiotic surfaces are preserved in interactions with living tissue.

Root exudates emerged as a key differentiator between abiotic surfaces and root tissue. Root-conditioned medium stimulated *gac* signaling, c-di-GMP production, and adhesion in a *gac-*dependent manner. While we do not yet know the exact exudate compound(s) responsible for this effect, our findings underscore how *gac* phenotypes seem to be particularly sensitive to chemical changes. Perhaps tuning the system to subtle chemical changes potentiates tissue colonization. More broadly, exudates are largely viewed as chemoattractants that promote motility toward the root (14). Our data support an expanded view whereby exudates also function as local signals that directly enhance root attachment (71). This finding underscores a fundamental difference between abiotic and tissue surfaces. Living tissues modify the chemical environment through secretion in a way that cannot be reproduced on abiotic substrates, and we propose that the *gac* system among *Pseudomonas fluorescens* species complex strains has evolved to incorporate chemical cues from roots into the biofilm activation program.

Two-component and phosphorelay systems frequently operate as signal-integration nodes by enforcing coincident detection of multiple signals before committing to irreversible responses (72–74). Our data place *gacSA* within this framework as a potential integrator of both chemical and physical cues from roots. While many studies have examined how the sensor kinase GacS is activated, there is still no consensus on the activating ligand(s) (75). Our data show that both surface contact sensing through the flagella and chemical cues in exudates activate the *gac* system on roots, but we do not yet know if these are direct activators of GacS. GacS activation triggers *gacA*-dependent expression of *rsm*s RNAs that go on to affect genes for biofilm formation, plant growth promotion, siderophore production, and antimicrobial production (39, 76, 77). Our data indicate that root attachment requires a *gac*-dependent increase in c-di-GMP levels, and we indeed identified two c-di-GMP metabolic genes that promote root attachment in our screen (*PFL_4532* and *PFL_5779*; Fig. 3). c-di-GMP binds transcriptional regulators that shift cells to a sessile state by repressing motility while activating adhesins and exopolysaccharides (78–80). The absence of strong hits in specific adhesins or exopolysaccharides in our screen likely reflects redundancy among attachment factors and suggests that regulation occurs primarily at the level of c-di-GMP signaling rather than direct transcriptional activation by *rsm* regulators. In many bacteria, surface colonization follows a relatively simple pathway in which surface contact elevates c-di-GMP and activates adhesins and biofilm matrix components (81, 82). In contrast, in the *Pseudomonas fluorescens* species complex, this transition is embedded within the global *gac* regulatory system. For example, in *Pseudomonas putida* KT2440, *gacA* mutants have low levels of c-di-GMP, reduced biofilm formation, and reduced expression of the *lapA* adhesin (55, 83). In *Pseudomonas ogarae* F113, the *gacSA* pathway converges with c-di-GMP signaling to control flagellar synthesis and motility (84). We extend these observations by identifying potential upstream inputs into this regulatory network. Our results suggest that this expanded regulation reflects the complexity of the rhizosphere, where root colonization likely requires coordinated activation of adhesion together with traits involved in competition and plant interaction (Fig. 6B). An important question is whether *gac* activation engages these programs proportionally or can bias them toward distinct outputs. One possibility is that differences in the magnitude or duration of *gac* signaling are encoded through the kinetics of the *rsm* sRNAs, allowing the *gac* system to tune the balance between adhesion and competitive behaviors depending on environmental conditions during root colonization.

Treatments with beneficial rhizobacteria perform poorly under field conditions in part because competition from native microbiota limits effective root colonization (85, 86). This ecological constraint means that even large inoculum doses or recurrent applications often provide limited long-term enhancement of plant performance. Current efforts to enhance colonization focus largely on improving delivery through strategies that position bacteria near emerging roots and by isolating new strains that may be better suited for competing in the rhizosphere. While these approaches can improve early establishment, they do not directly address how bacteria transition from bulk soil to the root surface. By showing that flagellar surface sensing stimulates the *gac* signaling system, our work suggests a new route to design more persistent bioinoculants. Rather than relying on genetic engineering or reformulating products, it could be possible to select for naturally occurring variants with reduced flagellar synthesis under the hypothesis that reduced motility will enhance root attachment at high inoculation titers. Although motility is important for reaching roots in soil, the lack of motility in such strains may not represent a disadvantage in applications where bacteria are directly delivered onto seeds or roots through technologies such as seed coatings. Under these conditions, enhanced early attachment could potentially yield inoculants that deliver more consistent yield enhancement in the field.

## MATERIALS AND METHODS

### Growth conditions and genetic manipulations

Growth media were supplemented with 1.5% (wt/vol) agar, 300 µM diaminopimelic acid (DAP), 50 µg/mL gentamycin, 100 µg/mL carbenicillin, and 50 µg/mL kanamycin when necessary. *E. coli* was cultured in LB medium at 37°C, *P. protegens* in King’s B medium (KBM) at 30°C, and *A. rhizogenes* in Nutrient broth at 30°C. Plasmids were introduced into Pf-5 by biparental conjugation with *E. coli* WM3064 as the donor strain. Gene deletions or insertions were generated using a two-step approach with a *sacB-*based counterselection. For gene deletions, two approximately 500 bp homology arms corresponding to the regions immediately upstream and downstream of the target gene were fused by Gibson assembly and inserted into the pDH100 plasmid. The upstream homology arm included the first 12 bp of the coding sequence, and the downstream homology arm included the last 12 bp of the coding sequence, creating an in-frame deletion designed to prevent polar effects on neighboring genes. For insertions, the DNA fragment to be introduced was inserted between approximately 500 bp upstream and downstream homology arms corresponding to the intended insertion site. WM3064 cells carrying the constructed pDH100 plasmids were mixed in a 1:1 ratio with Pf-5, spotted on LB medium supplemented with DAP and no antibiotics, and incubated at 30°C for 24 h to allow conjugation. Cells were then scraped from the conjugation plate, plated on LB agar supplemented with kanamycin, and incubated until kanamycin-resistant primary integrants were obtained. A single integrant colony was inoculated into LB broth and grown for 24 h without selection to allow for a second recombination event. Cultures were then plated on TYS20 agar (10 g/L tryptone, 5 g/L yeast extract, and 20% [wt/vol] sucrose) to select for double-crossover recombinants. Mutants were complemented by integrating the deleted gene driven by an arabinose-inducible promoter at the chromosomal Tn7 attachment site. The complementation construct was cloned into pUC18T-mini-Tn7T, and this plasmid and the helper plasmid pJMP1039 were separately introduced into WM3064. Donor strains carrying each plasmid were mixed with Pf-5 in a 1:1:1 ratio and plated on gentamicin to select for transconjugants (87). Mutants were fluorescently tagged by inserting an mVenus expression cassette into the CTX attachment site or the Tn7 attachment site (87, 88). Plasmids, strains, and primers used in this study are listed in Tables S2, S3, and S4, respectively.

### Establishment of tomato hairy root cultures

Tomato (*Solanum lycopersicum* cv. Rio-Grande) seedlings were transformed with *Agrobacterium rhizogenes* 15834 to generate hairy roots as previously described (89). To generate root biomass, the most vigorous roots, identified by their bright white color, high density of root hairs, and length, were excised and transferred to 250 mL flasks containing 50 mL of 0.5 × MS medium (pH 5.8) with 3% sucrose and supplemented with 200 µg/mL carbenicillin and 200 µg/mL cefotaxime to suppress *Agrobacterium* growth. Flasks were incubated in the dark at 25°C with shaking at 75 rpm, and the medium was refreshed weekly. After 2-3 weeks, when roots had filled the entire flask volume, they were cut and transferred into microplate wells for attachment assays.

### Tn-*Himar* mutant library construction and mapping

Construction and mapping of the Pf-5 transposon library was performed based on the method developed by Wetmore et al. (36). Mid-log phase cells from cultures of the APA_752 barcoded transposon pool grown in LB medium supplemented with kanamycin and DAP and mid-log phase Pf-5 cells from cultures grown in KBM were collected by centrifugation, washed, mixed in a 1:1 ratio, and spotted on a KBM agar plate containing DAP. After incubation for 24 h at 30°C, the cells were scraped from the plate, resuspended in KBM medium, and spread onto 20 150-mm KBM plates containing kanamycin, followed by incubation at 30°C for 3 days. Colonies were scraped into KBM medium and used to inoculate a KBM culture containing 25 μg/mL kanamycin. The culture was grown for three doublings, glycerol was added to 15%, and 1 mL aliquots were frozen at −80°C. Mapping genomic insertion positions was performed as described previously (69). The features of the obtained library are as follows: 57,304,857 total reads, 25,081,887 mapped reads in pool, 784,810 mapped barcodes in pool, 161,378 distinct insertion sites, 5,816 coding genes with central insertions, 53 median strains per hit protein, and 81.7 mean strains per hit protein.

### Adhesion profiling of barcoded Tn-*Himar* mutant libraries

Adhesion profiling was performed based on the method developed by Hershey et al. (69). Flasks containing hairy roots were prepared by refreshing the medium 48 h prior to inoculation, allowing root-secreted compounds to accumulate. A 750 μL inoculum of the barcoded transposon library was added to a 250 mL flask containing 75 mL of medium and hairy roots, and flasks were incubated for 24 h at 25°C with shaking at 75 rpm to allow bacterial attachment. Following incubation, 750 μL of the culture was transferred into a new flask with hairy roots, while cells from an additional 30 mL of culture were collected by centrifugation and stored at −20°C for BarSeq analysis. This procedure was performed five times for a total of five sequential passaging rounds. Parallel cultures were grown in flasks containing root-conditioned medium but no hairy roots, serving as a reference condition. Genomic DNA was extracted from cell pellets and served as templates for amplifying the barcodes in each sample using indexed primers (36). Amplicons were purified using AMPure XP magnetic beads (Beckman Coulter) and pooled for multiplexed sequencing. Next, 50-bp single-end reads were collected on an Illumina NovaSeq X Plus sequencer. MultiCodes.pl, combineBarSeq.pl, and BarSeqR.pl were used to determine fitness by comparing the log_2_ ratios of barcode counts in each sample over the counts from nonselective growth without hairy roots. For each mutant, the pracma R package was used to calculate the area under the curve of fitness scores across passages to rank mutants according to their hypo- or hyper-adhesive phenotypes.

### Crystal violet staining assay

Overnight cultures were resuspended in 0.5 × MS medium (pH 5.8) supplemented with 3% sucrose and 20 mM succinate, which was either freshly prepared or conditioned with hairy roots for 24 h. Resuspensions were normalized to OD_600_ 0.002, and 450 μL was added to each well of a 48-well microtiter plate. Plates were incubated shaking for 24 h at 30°C and 200 rpm. OD_600_ was measured to account for growth differences. Culture broth was discarded, and the wells were washed by dipping the microtiter plate in a bin with tap water; 500 μL of 0.1% (wt/vol) crystal violet solution was added to each well, and the plate was shaken for 30 min before the dye was discarded. The wells were washed in the same manner. The retained dye was dissolved in 500 μL of absolute ethanol by shaking for 10 min. OD_575_ was measured in each well and normalized to OD_600_.

### Measurement of c-di-GMP

Strains were transformed with the pConRef-2H12.D11 c-di-GMP reporter plasmid (41). Overnight cultures were resuspended in 0.5 × MS medium (pH 5.8) supplemented with 3% sucrose and 20 mM succinate. MS media were either freshly prepared or conditioned with hairy roots for 24 h. Cell suspensions were normalized to OD_600_ 0.02, and 200 μL was dispensed into wells of a 96-well microtiter plate. Plates were incubated shaking for 24 h at 30°C and 200 rpm, after which fluorescence was measured using a BioTek Synergy H1 microplate reader for cdGreen2.1 (excitation: 470 nm; emission: 515 nm) and normalized to the reference mScarlet-I (excitation: 575 nm; emission: 632 nm).

### Measurement of *gac* activity reporters

Strains were grown overnight in 0.5× MS medium (pH 5.8) supplemented with 3% sucrose and 20 mM succinate. To evaluate the effects of the Δ*fliF* mutation and methyl cellulose on reporter activity, overnight cultures were diluted 100-fold into 96-well plates containing 200 μL of the indicated medium per well and incubated for 24 h. Plates were shaken when assessing the effect of the Δ*fliF* mutation and incubated statically for methyl cellulose. Fluorescence was measured using a BioTek Synergy H1 microplate reader (mVenus; excitation: 500 nm; emission: 540 nm) and normalized to OD_600_. For microscopy, overnight cultures were diluted 10-fold, and 2 µL of cells was placed on a 2% agarose pad to immobilize the cells. Cells were imaged immediately to quantify reporter activity in planktonic cells and imaged again after 1 h to measure reporter activity in surface-sensing cells. Microscopy was performed using a Nikon Ti-E-inverted microscope equipped with an Orca Fusion BT digital CMOS camera (Hamamatsu). Fluorescence images were collected using a Prior Lumen 200 metal halide light source and a YFP-specific filter set (Chroma) with identical exposure times and laser intensity. Image analysis was performed with Fiji software.

### Root attachment assays

Hairy roots were excised into segments and placed in wells of a 48-well plate with 0.5 × MS medium (pH 5.8) supplemented with 3% sucrose and 20 mM succinate conditioned by roots for 24 h. Wells were inoculated with the indicated strains at an OD_600_ of 0.02. Plates were incubated at 25 °C with shaking at 75 rpm. To qualitatively assess bacterial root attachment, roots were washed three times within the plate wells and imaged for fluorescence using the Invitrogen iBright CL1500 Imaging System (excitation: 455–485 nm, emission: 508–557 nm). To quantitatively assess root attachment, roots were transferred to microcentrifuge tubes containing 1 mL of 0.5 × MS medium. Root-associated bacteria were detached by vortexing for 1 min, and the resulting suspensions were serially diluted and plated on LB agar for CFU enumeration, or had their fluorescence measured using a BioTek Synergy H1 microplate reader (mVenus; excitation: 500 nm; emission: 540 nm).

### Bacterial growth curve measurement

Overnight cultures were grown overnight in LB medium at 30°C and adjusted to an OD_600_ of 0.02 in indicated media; 200 µL was dispensed into 96-well plates, with five technical replicates per strain. Plates were incubated at 30°C with shaking in a BioTek Synergy H1 microplate reader, and OD_600_ was recorded at 30-min intervals for the duration of the experiment.

## Data Availability

The sequence data used to map the barcoded Tn-Himar library and the barcoded amplicon sequences collected after passaging in plant roots were deposited in the NCBI Sequence Read Archive (SRA) under BioProject number PRJNA1440512. Strains, plasmids, and details of their construction are available upon request.

## SUPPLEMENTAL MATERIAL

Supplemental material aem.00778-26-s0001.pdf Fig. S1 to S5; Tables S2 to S4.

Table S1 aem.00778-26-s0002.xlsx Tn-Seq fitness scores.

ASM does not own the copyrights to Supplemental Material that may be linked to, or accessed through, an article. The authors have granted ASM a non-exclusive, world-wide license to publish the Supplemental Material files. Please contact the corresponding author directly for reuse.

## References

[1] Sauer K, Stoodley P, Goeres DM, Hall-Stoodley L, Burmølle M, Stewart PS, Bjarnsholt T. 2022. The biofilm life cycle: expanding the conceptual model of biofilm formation. Nat Rev Microbiol 20:608–620. doi:10.1038/s41579-022-00767-0 35922483 PMC9841534

[2] Flemming H-C, Wuertz S. 2019. Bacteria and archaea on earth and their abundance in biofilms. Nat Rev Microbiol 17:247–260. doi:10.1038/s41579-019-0158-9 30760902

[3] Baxter KJ, Sas E, Clark KB, Walsh M, Pradeep N, Batool A, Naney C, Vargas Cruz MA, Kennerdale N, Das K, Shi Z, Kelam A, Verma V, Simões MF, Neefs D, Ravichandran V, Tirumalai MR, Barcenilla BB, Macori G, Gonzalez E, Sikes B, Karouia F, Brereton NJB. 2026. Biofilms: from the cradle of life to life support. NPJ Biofilms Microbiomes 12:11. doi:10.1038/s41522-025-00875-8 41571651 PMC12828010

[4] Grari O, Ezrari S, El Yandouzi I, Benaissa E, Ben Lahlou Y, Lahmer M, Saddari A, Elouennass M, Maleb A. 2025. A comprehensive review on biofilm-associated infections: mechanisms, diagnostic challenges, and innovative therapeutic strategies. The Microbe 8:100436. doi:10.1016/j.microb.2025.100436

[5] Liu Y, Shi A, Chen Y, Xu Z, Liu Y, Yao Y, Wang Y, Jia B. 2025. Beneficial microorganisms: regulating growth and defense for plant welfare. Plant Biotechnol J 23:986–998. doi:10.1111/pbi.14554 39704146 PMC11869181

[6] Rumbaugh KP, Whiteley M. 2025. Towards improved biofilm models. Nat Rev Microbiol 23:57–66. doi:10.1038/s41579-024-01086-2 39112554

[7] Huang R, Li M, Gregory RL. 2011. Bacterial interactions in dental biofilm. Virulence 2:435–444. doi:10.4161/viru.2.5.16140 21778817 PMC3322631

[8] Kolenbrander PE, London J. 1993. Adhere today, here tomorrow: oral bacterial adherence. J Bacteriol 175:3247–3252. doi:10.1128/jb.175.11.3247-3252.1993 8501028 PMC204720

[9] Torres M, Paszti S, Eberl L. 2024. Shedding light on bacteria-host interactions with the aid of TnSeq approaches. mBio 15:e0039024. doi:10.1128/mbio.00390-24 38722161 PMC11237515

[10] Pearce D, Bazin MJ, Lynch JM. 1995. The rhizosphere as a biofilm, p 207–220. *In* Lappin-Scott HM, Costerton JW (ed), Microbial Biofilms. Cambridge University Press, Cambridge.

[11] Liu Y, Xu Z, Chen L, Xun W, Shu X, Chen Y, Sun X, Wang Z, Ren Y, Shen Q, Zhang R. 2024. Root colonization by beneficial rhizobacteria. FEMS Microbiol Rev 48:fuad066. doi:10.1093/femsre/fuad066 38093453 PMC10786197

[12] Bhattacharyya A, Mavrodi O, Bhowmik N, Weller D, Thomashow L, Mavrodi D. 2023. Bacterial biofilms as an essential component of rhizosphere plant-microbe interactions. Methods Microbiol 53:3–48. doi:10.1016/bs.mim.2023.05.006 38415193 PMC10898258

[13] Robert CAM, Himmighofen P, McLaughlin S, Cofer TM, Khan SA, Siffert A, Sasse J. 2025. Environmental and biological drivers of root exudation. Annu Rev Plant Biol 76:317–339. doi:10.1146/annurev-arplant-083123-082752 40063678

[14] Yang C-X, Chen S-J, Hong X-Y, Wang L-Z, Wu H-M, Tang Y-Y, Gao Y-Y, Hao G-F. 2025. Plant exudates-driven microbiome recruitment and assembly facilitates plant health management. FEMS Microbiol Rev 49:fuaf008. doi:10.1093/femsre/fuaf008 40158196 PMC12007450

[15] Liu Z, Beskrovnaya P, Melnyk RA, Hossain SS, Khorasani S, O’Sullivan LR, Wiesmann CL, Bush J, Richard JD, Haney CH. 2018. A genome-wide screen identifies genes in rhizosphere-associated Pseudomonas required to evade plant defenses. mBio 9:e00433-18. doi:10.1128/mBio.00433-18 30401768 PMC6222131

[16] Torres M, Jiquel A, Jeanne E, Naquin D, Dessaux Y, Faure D. 2022. Agrobacterium tumefaciens fitness genes involved in the colonization of plant tumors and roots. New Phytol 233:905–918. doi:10.1111/nph.17810 34655498

[17] do Amaral FP, Tuleski TR, Pankievicz VCS, Melnyk RA, Arkin AP, Griffitts J, Tadra-Sfeir MZ, Maltempi de Souza E, Deutschbauer A, Monteiro RA, Stacey G. 2020. Diverse bacterial genes modulate plant root association by beneficial bacteria. mBio 11:e03078-20. doi:10.1128/mBio.03078-20 PMC7774000 33323518

[18] Cole BJ, Feltcher ME, Waters RJ, Wetmore KM, Mucyn TS, Ryan EM, Wang G, Ul-Hasan S, McDonald M, Yoshikuni Y, Malmstrom RR, Deutschbauer AM, Dangl JL, Visel A. 2017. Genome-wide identification of bacterial plant colonization genes. PLoS Biol 15:e2002860. doi:10.1371/journal.pbio.2002860 28938018 PMC5627942

[19] Knights HE, Ramachandran VK, Jorrin B, Ledermann R, Parsons JD, Aroney STN, Poole PS. 2024. Rhizobium determinants of rhizosphere persistence and root colonization. ISME J 18:wrae072. doi:10.1093/ismejo/wrae072 38690786 PMC11103875

[20] Torres M, Price MN, Khasanova A, Kosina SM, Zhalnina K, Northen TR, Deutschbauer AM. 2025. Bacterial fitness for plant colonization is influenced by plant growth substrate. New Phytol 248:3168–3190. doi:10.1111/nph.70617 41025825 PMC12630448

[21] Sivakumar R, Ranjani J, Vishnu US, Jayashree S, Lozano GL, Miles J, Broderick NA, Guan C, Gunasekaran P, Handelsman J, Rajendhran J. 2019. Evaluation of INSeq to identify genes essential for Pseudomonas aeruginosa PGPR2 corn root colonization. G3 (Bethesda) 9:651–661. doi:10.1534/g3.118.200928 30705119 PMC6404608

[22] Ghaly TM, Fabian BK, Vick SHW, Foster C, Asher AJ, Hassan KA, Elbourne LDH, Paulsen IT, Tetu SG. 2025. Genetic drivers of plant root colonisation by the biocontrol agent Pseudomonas protegens Pf-5. Environ Microbiol Rep 17:e70179. doi:10.1111/1758-2229.70179 40827064 PMC12361813

[23] Cheng X, Etalo DW, van de Mortel JE, Dekkers E, Nguyen L, Medema MH, Raaijmakers JM. 2017. Genome-wide analysis of bacterial determinants of plant growth promotion and induced systemic resistance by Pseudomonas fluorescens. Environ Microbiol 19:4638–4656. doi:10.1111/1462-2920.13927 28892231

[24] Pranav PS, Sivakumar R, Suvekbala V, Rajendhran J. 2024. Genome-wide identification of root colonization fitness genes in plant growth promoting Pseudomonas asiatica employing transposon-insertion sequencing. Ann Microbiol 74:40. doi:10.1186/s13213-024-01784-5

[25] Taylor TB, Silby MW, Jackson RW. 2025. Pseudomonas fluorescens. Trends Microbiol 33:250–251. doi:10.1016/j.tim.2024.11.005 39613688

[26] Zboralski A, Filion M. 2020. Genetic factors involved in rhizosphere colonization by phytobeneficial Pseudomonas spp. Comput Struct Biotechnol J 18:3539–3554. doi:10.1016/j.csbj.2020.11.025 33304453 PMC7711191

[27] Krell T, Matilla MA. 2024. Pseudomonas aeruginosa*.* Trends Microbiol 32:216–218. doi:10.1016/j.tim.2023.11.005 38065787

[28] Collins AJ, Smith TJ, Sondermann H, O’Toole GA. 2020. From input to output: the Lap/c-di-GMP biofilm regulatory circuit. Annu Rev Microbiol 74:607–631. doi:10.1146/annurev-micro-011520-094214 32689917 PMC8966053

[29] Loper JE, Kobayashi DY, Paulsen IT. 2007. The genomic sequence of Pseudomonas fluorescens Pf-5: insights into biological control. Phytopathology 97:233–238. doi:10.1094/PHYTO-97-2-0233 18944380

[30] Paulsen IT, Press CM, Ravel J, Kobayashi DY, Myers GSA, Mavrodi DV, DeBoy RT, Seshadri R, Ren Q, Madupu R, et al.. 2005. Complete genome sequence of the plant commensal Pseudomonas fluorescens Pf-5. Nat Biotechnol 23:873–878. doi:10.1038/nbt1110 15980861 PMC7416659

[31] Jing X, Cui Q, Li X, Yin J, Ravichandran V, Pan D, Fu J, Tu Q, Wang H, Bian X, Zhang Y. 2020. Engineering Pseudomonas protegens Pf-5 to improve its antifungal activity and nitrogen fixation. Microb Biotechnol 13:118–133. doi:10.1111/1751-7915.13335 30461205 PMC6984399

[32] Zhao Q, Wang R, Song Y, Lu J, Zhou B, Song F, Zhang L, Huang Q, Gong J, Lei J, Dong S, Gu Q, Borriss R, Gao X, Wu H. 2024. Pyoluteorin-deficient Pseudomonas protegens improves cooperation with Bacillus velezensis, biofilm formation, co-colonizing, and reshapes rhizosphere microbiome. NPJ Biofilms Microbiomes 10:145. doi:10.1038/s41522-024-00627-0 39663366 PMC11634903

[33] Ron M, Kajala K, Pauluzzi G, Wang D, Reynoso MA, Zumstein K, Garcha J, Winte S, Masson H, Inagaki S, Federici F, Sinha N, Deal RB, Bailey-Serres J, Brady SM. 2014. Hairy root transformation using Agrobacterium rhizogenes as a tool for exploring cell type-specific gene expression and function using tomato as a model. Plant Physiol 166:455–469. doi:10.1104/pp.114.239392 24868032 PMC4213079

[34] Biswas D, Chakraborty A, Mukherjee S, Ghosh B. 2023. Hairy root culture: a potent method for improved secondary metabolite production of Solanaceous plants. Front Plant Sci 14:1197555. doi:10.3389/fpls.2023.1197555 37731987 PMC10507345

[35] Kamilova F, Kravchenko LV, Shaposhnikov AI, Makarova N, Lugtenberg B. 2006. Effects of the tomato pathogen Fusarium oxysporum f. sp. radicis-lycopersici and of the biocontrol bacterium Pseudomonas fluorescens WCS365 on the composition of organic acids and sugars in tomato root exudate. Mol Plant Microbe Interact 19:1121–1126. doi:10.1094/MPMI-19-1121 17022176

[36] Wetmore KM, Price MN, Waters RJ, Lamson JS, He J, Hoover CA, Blow MJ, Bristow J, Butland G, Arkin AP, Deutschbauer A. 2015. Rapid quantification of mutant fitness in diverse bacteria by sequencing randomly bar-coded transposons. mBio 6:e00306-15. doi:10.1128/mBio.00306-15 25968644 PMC4436071

[37] Niehaus TD, Elbadawi-Sidhu M, de Crécy-Lagard V, Fiehn O, Hanson AD. 2017. Discovery of a widespread prokaryotic 5-oxoprolinase that was hiding in plain sight. J Biol Chem 292:16360–16367. doi:10.1074/jbc.M117.805028 28830929 PMC5625064

[38] Laventie B-J, Jenal U. 2020. Surface sensing and adaptation in bacteria. Annu Rev Microbiol 74:735–760. doi:10.1146/annurev-micro-012120-063427 32905753

[39] Song H, Li Y, Wang Y. 2023. Two-component system GacS/GacA, a global response regulator of bacterial physiological behaviors. Eng Microbiol 3:100051. doi:10.1016/j.engmic.2022.100051 39628522 PMC11611043

[40] Dufrêne YF, Persat A. 2020. Mechanomicrobiology: how bacteria sense and respond to forces. Nat Rev Microbiol 18:227–240. doi:10.1038/s41579-019-0314-2 31959911

[41] Kaczmarczyk A, van Vliet S, Jakob RP, Teixeira RD, Scheidat I, Reinders A, Klotz A, Maier T, Jenal U. 2024. A genetically encoded biosensor to monitor dynamic changes of c-di-GMP with high temporal resolution. Nat Commun 15:3920. doi:10.1038/s41467-024-48295-0 38724508 PMC11082216

[42] Kay E, Dubuis C, Haas D. 2005. Three small RNAs jointly ensure secondary metabolism and biocontrol in Pseudomonas fluorescens CHA0. Proc Natl Acad Sci USA 102:17136–17141. doi:10.1073/pnas.0505673102 16286659 PMC1287983

[43] Zha D, Xu L, Zhang H, Yan Y. 2014. The two-component GacS-GacA system activates lipA translation by RsmE but not RsmA in Pseudomonas protegens Pf-5. Appl Environ Microbiol 80:6627–6637. doi:10.1128/AEM.02184-14 25128345 PMC4249036

[44] Pijper A. 1947. Methylcellulose and bacterial motility. J Bacteriol 53:257–269. doi:10.1128/jb.53.3.257-269.1947 16561270 PMC518306

[45] Joller C, Waelchli J, Schlaepfer J, Schlaeppi K. 2026. Paralleled dynamics of arabidopsis root exudation and SynCom assembly in a controlled environment. Plant Biology. doi:10.64898/2026.01.29.702624

[46] McLaughlin S, Zhalnina K, Kosina S, Northen TR, Sasse J. 2023. The core metabolome and root exudation dynamics of three phylogenetically distinct plant species. Nat Commun 14:1649. doi:10.1038/s41467-023-37164-x 36964135 PMC10039077

[47] Lozano GL, Bravo JI, Garavito Diago MF, Park HB, Hurley A, Peterson SB, Stabb EV, Crawford JM, Broderick NA, Handelsman J. 2019. Introducing THOR, a model microbiome for genetic dissection of community behavior. mBio 10:e02846-18. doi:10.1128/mBio.02846-18 PMC6401489 30837345

[48] Wallner A, Busset N, Lachat J, Guigard L, King E, Rimbault I, Mergaert P, Béna G, Moulin L. 2022. Differential genetic strategies of Burkholderia vietnamiensis and Paraburkholderia kururiensis for root colonization of Oryza sativa subsp. japonica and O. sativa subsp. indica, as revealed by transposon mutagenesis sequencing. Appl Environ Microbiol 88:e0064222. doi:10.1128/aem.00642-22 35862731 PMC9317867

[49] López-Sánchez A, Leal-Morales A, Jiménez-Díaz L, Platero AI, Bardallo-Pérez J, Díaz-Romero A, Acemel RD, Illán JM, Jiménez-López J, Govantes F. 2016. Biofilm formation-defective mutants in Pseudomonas putida. FEMS Microbiol Lett 363:fnw127. doi:10.1093/femsle/fnw127 27190143

[50] Zhang L, Shi Y, Wu Z, Tan G. 2018. Characterization of response regulator GacA involved in phaseolotoxin production, hypersensitive response and cellular processes in Pseudomonas syringae pv. actinidiae A18. Physiol Mol Plant Pathol 103:137–142. doi:10.1016/j.pmpp.2018.07.001

[51] Takeuchi K. 2018. GABA, a primary metabolite controlled by the Gac/Rsm regulatory pathway, favors a planktonic over a biofilm lifestyle in Pseudomonas protegens CHA0. Mol Plant Microbe Interact 31:274–282. doi:10.1094/MPMI-05-17-0120-R 28990487

[52] Marutani M, Taguchi F, Ogawa Y, Hossain MM, Inagaki Y, Toyoda K, Shiraishi T, Ichinose Y. 2008. Gac two-component system in Pseudomonas syringae pv. tabaci is required for virulence but not for hypersensitive reaction. Mol Genet Genomics 279:313–322. doi:10.1007/s00438-007-0309-y 18080141

[53] Kidarsa TA, Shaffer BT, Goebel NC, Roberts DP, Buyer JS, Johnson A, Kobayashi DY, Zabriskie TM, Paulsen I, Loper JE. 2013. Genes expressed by the biological control bacterium Pseudomonas protegens Pf-5 on seed surfaces under the control of the global regulators GacA and RpoS. Environ Microbiol 15:716–735. doi:10.1111/1462-2920.12066 23297839

[54] Lalaouna D, Fochesato S, Sanchez L, Schmitt-Kopplin P, Haas D, Heulin T, Achouak W. 2012. Phenotypic switching in Pseudomonas brassicacearum involves GacS- and GacA-dependent rsm small RNAs. Appl Environ Microbiol 78:1658–1665. doi:10.1128/AEM.06769-11 22247157 PMC3298130

[55] Martínez-Gil M, Ramos-González MI, Espinosa-Urgel M. 2014. Roles of cyclic Di-GMP and the gac system in transcriptional control of the genes coding for the Pseudomonas putida adhesins LapA and LapF. J Bacteriol 196:1484–1495. doi:10.1128/JB.01287-13 24488315 PMC3993364

[56] Driscoll WW, Pepper JW, Pierson LS, Pierson EA. 2011. Spontaneous gac mutants of Pseudomonas biological control strains: cheaters or mutualists? Appl Environ Microbiol 77:7227–7235. doi:10.1128/AEM.00679-11 21873476 PMC3194857

[57] Cheng X, de Bruijn I, van der Voort M, Loper JE, Raaijmakers JM. 2013. The gac regulon of Pseudomonas fluorescens SBW25. Environ Microbiol Rep 5:608–619. doi:10.1111/1758-2229.12061 23864577

[58] Luo Y, Srinivas A, Guidry C, Bull C, Haney CH, Hamilton C. 2025. GacA regulates symbiosis and mediates lifestyle transitions in Pseudomonas mSphere 10:e0027725. doi:10.1128/msphere.00277-25 40919919 PMC12482163

[59] Kim JS, Kim YH, Park JY, Anderson AJ, Kim YC. 2014. The global regulator GacS regulates biofilm formation in Pseudomonas chlororaphis O6 differently with carbon source. Can J Microbiol 60:133–138. doi:10.1139/cjm-2013-0736 24588386

[60] Li J, Yang Y, Dubern J-F, Li H, Halliday N, Chernin L, Gao K, Cámara M, Liu X. 2015. Regulation of GacA in Pseudomonas chlororaphis strains shows a niche specificity. PLoS One 10:e0137553. doi:10.1371/journal.pone.0137553 26379125 PMC4574860

[61] Natsch A, Keel C, Pfirter HA, Haas D, Défago G. 1994. Contribution of the global regulator gene gacA to persistence and dissemination of Pseudomonas fluorescens biocontrol strain CHA0 introduced into soil microcosms. Appl Environ Microbiol 60:2553–2560. doi:10.1128/aem.60.7.2553-2560.1994 16349332 PMC201683

[62] Zuber S, Carruthers F, Keel C, Mattart A, Blumer C, Pessi G, Gigot-Bonnefoy C, Schnider-Keel U, Heeb S, Reimmann C, Haas D. 2003. GacS sensor domains pertinent to the regulation of exoproduct formation and to the biocontrol potential of Pseudomonas fluorescens CHA0. Mol Plant Microbe Interact 16:634–644. doi:10.1094/MPMI.2003.16.7.634 12848429

[63] Song C, Kidarsa TA, van de Mortel JE, Loper JE, Raaijmakers JM. 2016. Living on the edge: emergence of spontaneous gac mutations in Pseudomonas protegens during swarming motility. Environ Microbiol 18:3453–3465. doi:10.1111/1462-2920.13288 26945503

[64] Li E, Zhang H, Jiang H, Pieterse CMJ, Jousset A, Bakker PAHM, de Jonge R. 2021. Experimental-evolution-driven identification of Arabidopsis rhizosphere competence genes in Pseudomonas protegens. mBio 12:e0092721. doi:10.1128/mBio.00927-21 34101491 PMC8262913

[65] Martínez-Granero F, Rivilla R, Martín M. 2006. Rhizosphere selection of highly motile phenotypic variants of Pseudomonas fluorescens with enhanced competitive colonization ability. Appl Environ Microbiol 72:3429–3434. doi:10.1128/AEM.72.5.3429-3434.2006 16672487 PMC1472367

[66] Chancey ST, Wood DW, Pierson EA, Pierson LS. 2002. Survival of GacS/GacA mutants of the biological control bacterium Pseudomonas aureofaciens 30-84 in the wheat rhizosphere. Appl Environ Microbiol 68:3308–3314. doi:10.1128/AEM.68.7.3308-3314.2002 12089008 PMC126771

[67] Ishizawa H, Kuroda M, Inoue D, Ike M. 2022. Genome-wide identification of bacterial colonization and fitness determinants on the floating macrophyte, duckweed. Commun Biol 5:68. doi:10.1038/s42003-022-03014-7 35046504 PMC8770550

[68] Li J, Zhang Y, Jiang W, Zhang L-Q. 2025. Experimental evolution of plant rhizobacteria reveals emerging adaptive mutations. mBio 16:e0102325. doi:10.1128/mbio.01023-25 40657900 PMC12345224

[69] Hershey DM, Fiebig A, Crosson S. 2021. Flagellar perturbations activate adhesion through two distinct pathways in Caulobacter crescentus. mBio 12:e03266-20. doi:10.1128/mBio.03266-20 33563824 PMC7885107

[70] Kuchma SL, Geiger CJ, Webster SS, Fu Y, Montoya R, O’Toole GA. 2025. Genetic analysis of flagellar-mediated surface sensing by Pseudomonas aeruginosa PA14. J Bacteriol 207:e0052024. doi:10.1128/jb.00520-24 40470954 PMC12288467

[71] O’Banion BS, Jones P, Demetros AA, Kelley BR, Knoor LH, Wagner AS, Chen J-G, Muchero W, Reynolds TB, Jacobson D, Lebeis SL. 2023. Plant myo-inositol transport influences bacterial colonization phenotypes. Current Biology 33:3111–3124. doi:10.1016/j.cub.2023.06.057 37419115

[72] Schultz D, Wolynes PG, Jacob EB, Onuchic JN. 2009. Deciding fate in adverse times: sporulation and competence in Bacillus subtilis*.* Proc Natl Acad Sci USA 106:21027–21034. doi:10.1073/pnas.0912185106 19995980 PMC2795487

[73] O’Malley MR, Dearing HN, Zheng X, Kretschmer AN, Cho TH-S, Raivio TL, Parsek MR. 2026. The Pseudomonas aeruginosa Cpx system provides a cyclic-di-GMP independent link between cell envelope stress and surface sensing. mBio 17:e0272625. doi:10.1128/mbio.02726-25 41400349 PMC12802237

[74] Mao M, He L, Yan Q. 2025. An updated overview on the bacterial PhoP/PhoQ two-component signal transduction system. Front Cell Infect Microbiol 15:1509037. doi:10.3389/fcimb.2025.1509037 39958932 PMC11825808

[75] Ali-Ahmad A, Fadel F, Sebban-Kreuzer C, Ba M, Pélissier GD, Bornet O, Guerlesquin F, Bourne Y, Bordi C, Vincent F. 2017. Structural and functional insights into the periplasmic detector domain of the GacS histidine kinase controlling biofilm formation in Pseudomonas aeruginosa. Sci Rep 7:11262. doi:10.1038/s41598-017-11361-3 28900144 PMC5595915

[76] Liang F, Zhang B, Yang Q, Zhang Y, Zheng D, Zhang L-Q, Yan Q, Wu X. 2020. Cyclic-di-GMP regulates the quorum-sensing system and biocontrol activity of Pseudomonas fluorescens 2P24 through the RsmA and RsmE proteins. Appl Environ Microbiol 86:e02016-20. doi:10.1128/AEM.02016-20 33036989 PMC7688223

[77] Zhang Y, Zhang B, Wu H, Wu X, Yan Q, Zhang L-Q. 2020. Pleiotropic effects of RsmA and RsmE proteins in Pseudomonas fluorescens 2P24. BMC Microbiol 20:191. doi:10.1186/s12866-020-01880-x 32615927 PMC7331252

[78] Collins AJ, Pastora AB, Smith TJ, O’Toole GA. 2020. MapA, a second large RTX Adhesin conserved across the Pseudomonads, contributes to biofilm formation by Pseudomonas fluorescens. J Bacteriol 202. doi:10.1128/JB.00277-20 PMC7925077 32631946

[79] Pastora AB, O’Toole GA. 2023. The regulator FleQ both transcriptionally and post-transcriptionally regulates the level of RTX adhesins of Pseudomonas fluorescens J Bacteriol 205:e0015223. doi:10.1128/jb.00152-23 37655913 PMC10521353

[80] Martínez-Granero F, Navazo A, Barahona E, Redondo-Nieto M, Rivilla R, Martín M. 2012. The Gac-Rsm and SadB signal transduction pathways converge on AlgU to downregulate motility in Pseudomonas fluorescens. PLoS One 7:e31765. doi:10.1371/journal.pone.0031765 22363726 PMC3282751

[81] Brinkley DM, Bertolli SK, Gallagher LA, Tan Y, Motha de Silva M, Brockman A, Zhang D, Peterson SB, Mougous JD. 2025. Pseudomonads coordinate innate defense against viruses and bacteria with a single regulatory system. Cell Host & Microbe 33:1333–1346. doi:10.1016/j.chom.2025.07.016 40812186 PMC12697246

[82] Liu C, Shi R, Jensen MS, Zhu J, Liu J, Liu X, Sun D, Liu W. 2024. The global regulation of c‐di‐GMP and cAMP in bacteria. mLife 3:42–56. doi:10.1002/mlf2.12104 38827514 PMC11139211

[83] Ramos-González MI, Travieso ML, Soriano MI, Matilla MA, Huertas-Rosales Ó, Barrientos-Moreno L, Tagua VG, Espinosa-Urgel M. 2016. Genetic dissection of the regulatory network associated with high c-di-GMP levels in Pseudomonas putida KT2440. Front Microbiol 7. doi:10.3389/fmicb.2016.01093 PMC4951495 27489550

[84] Muriel C, Blanco-Romero E, Trampari E, Arrebola E, Durán D, Redondo-Nieto M, Malone JG, Martín M, Rivilla R. 2019. The diguanylate cyclase AdrA regulates flagellar biosynthesis in Pseudomonas fluorescens F113 through SadB. Sci Rep 9:8096. doi:10.1038/s41598-019-44554-z 31147571 PMC6543031

[85] Azarbad H, Junker RR. 2024. Biological and experimental factors that define the effectiveness of microbial inoculation on plant traits: a meta-analysis. ISME Communications 4. doi:10.1093/ismeco/ycae122 PMC11538580 39507396

[86] Lopes M dos S, Dias-Filho MB, Gurgel ESC. 2021. Successful plant growth-promoting microbes: inoculation methods and abiotic factors. Front Sustain Food Syst 5. doi:10.3389/fsufs.2021.606454

[87] Gheorghita AA, Wolfram F, Whitfield GB, Jacobs HM, Pfoh R, Wong SSY, Guitor AK, Goodyear MC, Berezuk AM, Khursigara CM, Parsek MR, Howell PL. 2022. The Pseudomonas aeruginosa homeostasis enzyme AlgL clears the periplasmic space of accumulated alginate during polymer biosynthesis. J Biol Chem 298:101560. doi:10.1016/j.jbc.2021.101560 34990713 PMC8829089

[88] Wang Z, Xiong G, Lutz F. 1995. Site-specific integration of the phage ΦCTX genome into the Pseudomonas aeruginosa chromosome: characterization of the functional integrase gene located close to and upstream of attP. Molec Gen Genet 246:72–79. doi:10.1007/BF00290135 7823914

[89] Morcillo RJL, Zhao A, Tamayo-Navarrete MI, García-Garrido JM, Macho AP. 2020. Tomato root transformation followed by inoculation with Ralstonia solanacearum for straightforward genetic analysis of bacterial wilt disease. Plant Biology. doi:10.1101/646158 32225152

