# Supplementary material for: The gac system integrates physical and chemical cues to promote plant root attachment
Source: Appl Environ Microbiol. 2026 Jun 26;92(7):e00778-26. doi: 10.1128/aem.00778-26 (PMC13390462; doi:10.1128/aem.00778-26)
Supplement: Supplemental material — Fig. S1 to S5; Tables S2 to S4. [file aem.00778-26-s0001.pdf]

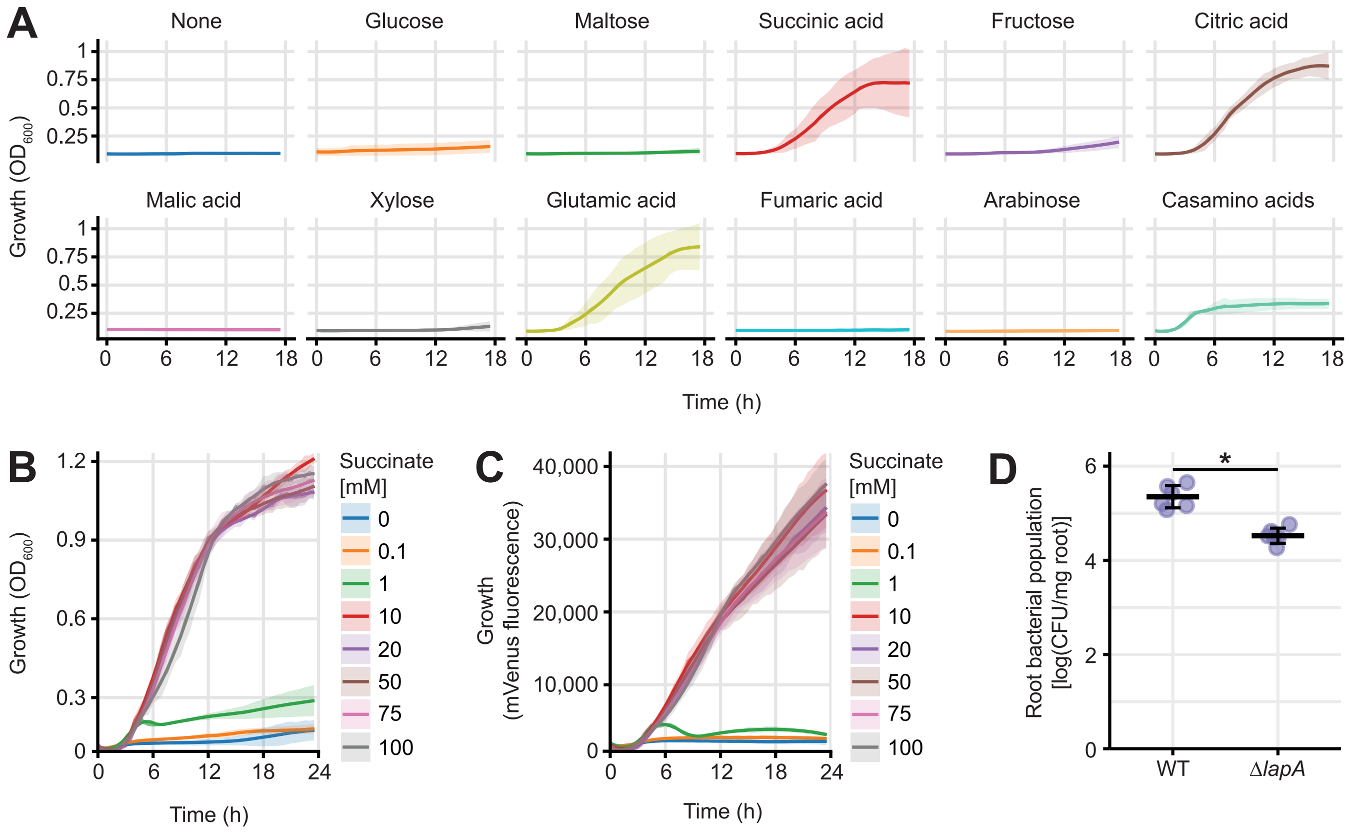

Figure S1. Optimization of root growth media for Pf-5 root colonization.

(A) Growth of mVenus-tagged Pf-5 in root growth media in the absence or addition of carbon sources commonly detected in root exudates. B and C, growth of mVenus-tagged Pf-5 in media supplemented with the indicated concentrations of succinate quantified by measuring OD_{600} (B) or mVenus fluorescence (C). (D) Root colonization phenotypes for wild-type and Δ*lapA* Pf-5 strains. Root colonization was quantified by recovering the root-attached fraction of cells and quantifying viable cell counts. A representative replicate of three independent experiments is shown. Data represent means ± SD. Statistical significance was determined by a pairwise *t*-test between wild-type and each strain. Asterisks indicate $P < 0.001$.

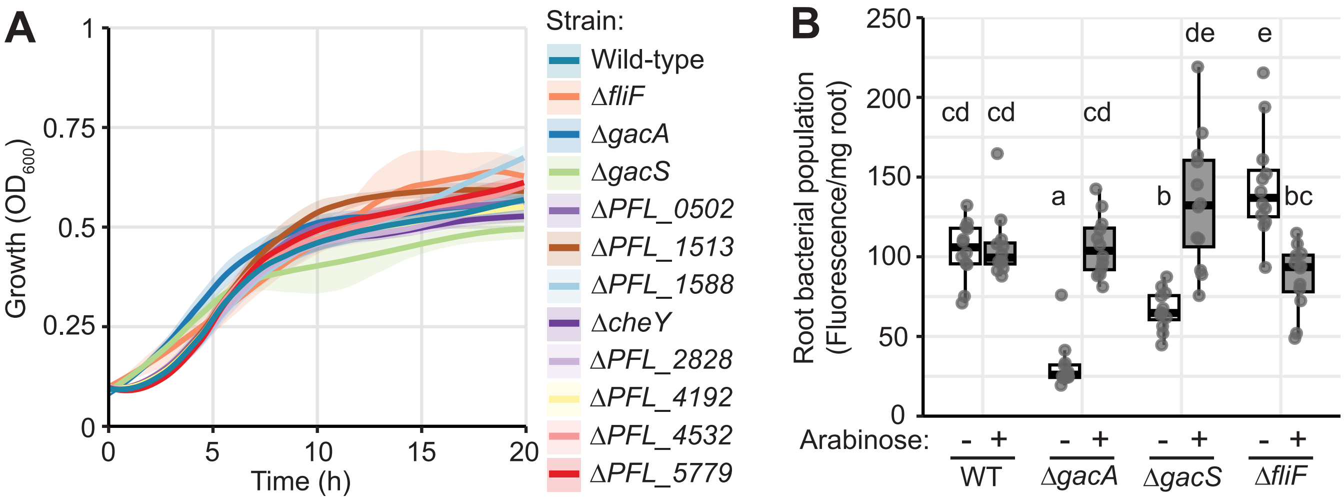

Figure S2. Growth analysis of deletion mutants and complementation of root colonization phenotypes.

(A) Growth of Pf-5 in-frame deletion mutants in root-conditioned media. (B) Root colonization was quantified by recovering the root-attached fraction of mVenus-tagged cells carrying a complementation construct expressed from an arabinose-inducible promoter and measuring emitted fluorescence. Where indicated, media was supplemented with 0.5% arabinose to induce gene expression. Bold lines within each box represent median values. Top and bottom sides of the boxes represent the third and first quartiles of the value distribution, respectively. Lines extending from the boxes denote the extreme values within 1.5 times the interquartile range. Data are pooled from three individual replicates. Different letters indicate statistically significant differences determined by two-way ANOVA with Tukey's post hoc test ($P < 0.05$).

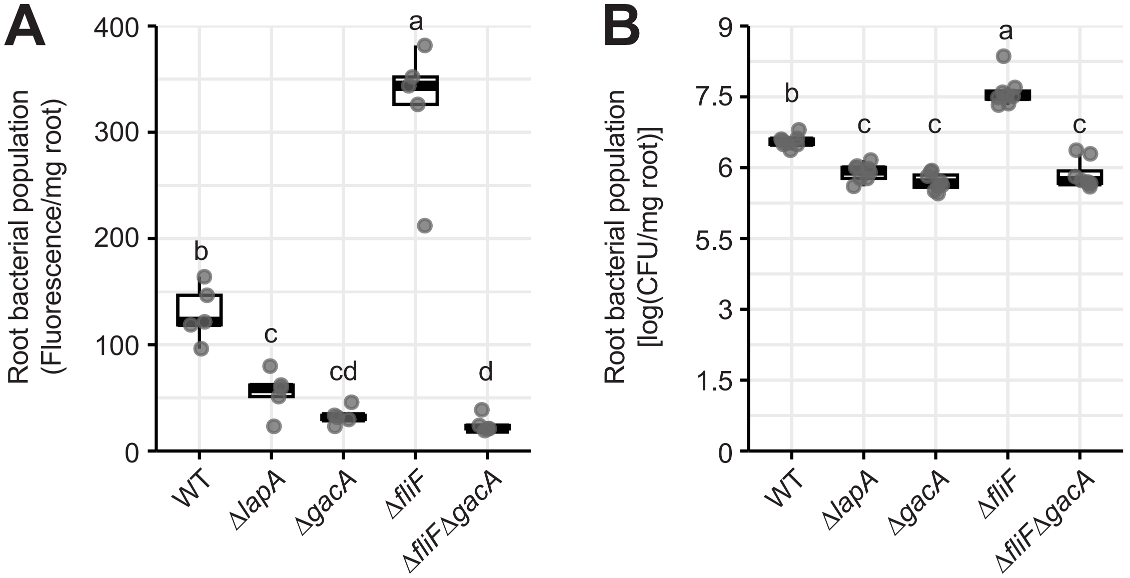

Figure S3. Root colonization phenotypes validation for in-frame deletion mutants.

Root colonization was quantified by recovering the root-attached fraction of mVenus-tagged cells inoculated onto 14-day-old seedlings and measuring emitted fluorescence (A), or by recovering the root-attached fraction of cells carrying a kanamycin resistance cassette from inoculated hairy roots and quantifying viable cell counts (B). Bold lines within each box represent median values. Top and bottom sides of the boxes represent the third and first quartiles of the value distribution, respectively. Lines extending from the boxes denote the extreme values within 1.5 times the interquartile range. Panels show a representative replicate of three individual experiments. Different letters indicate statistically significant differences determined by one-way ANOVA with Tukey’s post hoc test ($P < 0.05$).

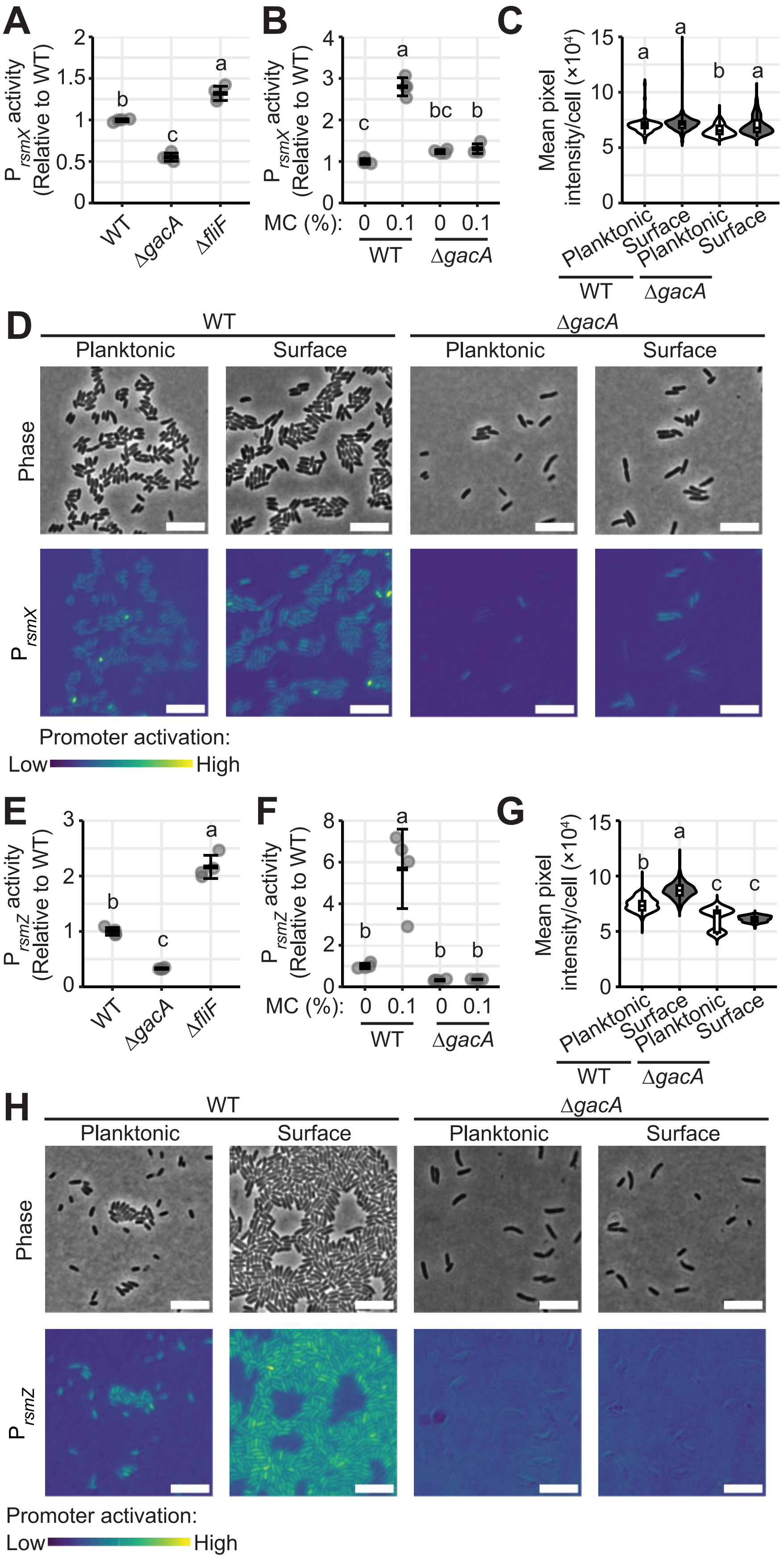

Figure S4. Flagellar-dependent surface sensing activates the *gac* system.

gac system activity was quantified using fluorescent transcriptional reporters for *rsmX* (A-D) or *rsmZ* (E-H). The effect of surface sensing on *gac* activity was evaluated by disrupting flagellar assembly (A and E), increasing medium viscosity with methyl cellulose (MC) (B and F), or comparing planktonic and surface-attached cells (C-D and G-H). In D and H, scale bars represent 5 μm. All panels show a representative replicate of three individual experiments. Data are means ± SD. In all panels letters represent statistical significance determined by one-way ANOVA and Tukey's posthoc test ($P < 0.05$).

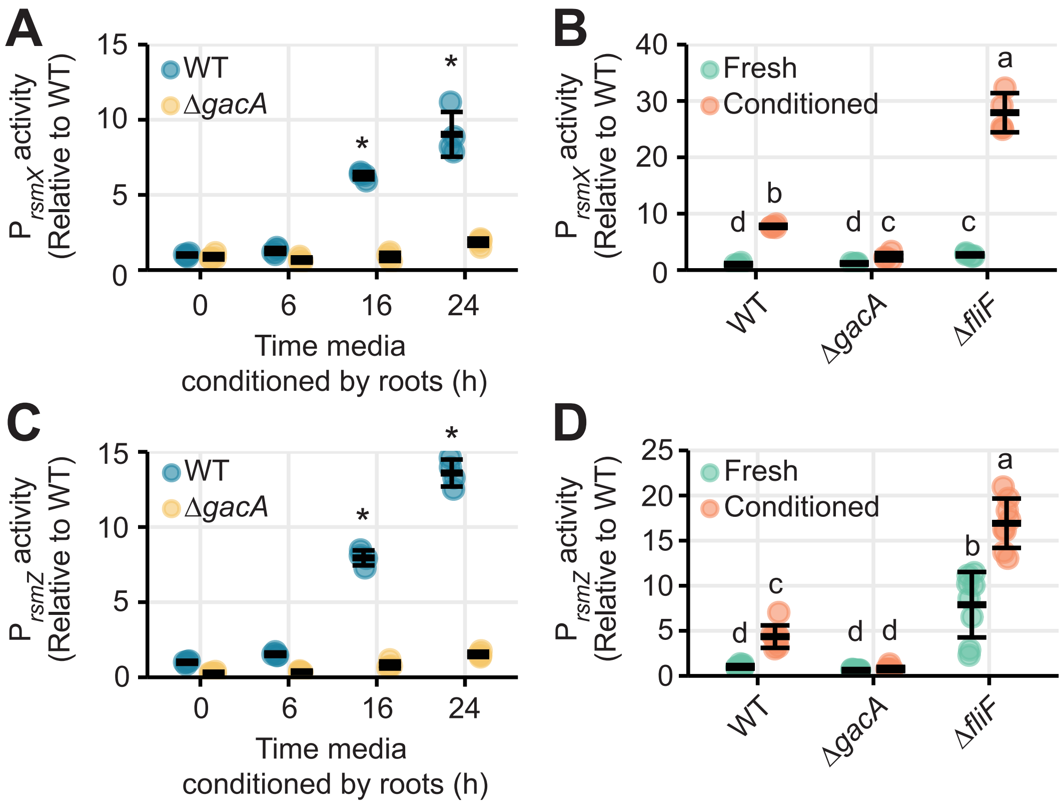

Figure S5. The *gac* system integrates physical and chemical cues to control surface attachment.

(A and C) Evaluating the effect of conditioning time of media by roots on *gac* system activity using *rsmX* (A) or *rsmZ* (C) fluorescent reporters. (B and D) Integration of chemical and mechanical inputs by the *gac* system. Strains were analyzed under conditions providing mechanical input alone (Δ*fliF*), chemical input alone (root-conditioned media), or both inputs combined. *gac* system activity was quantified via the *rsmX* (B) or the *rsmZ* (D) fluorescent reporters. *gac* system activity levels were normalized to wild-type in unconditioned (Fresh) media. Data represent means ± SD. In panels B and D, different letters indicate statistically significant differences determined by one-way ANOVA with Tukey's post hoc test ($P < 0.05$). In panel A and C, asterisks indicate statistically significant differences determined by pairwise *t*-tests comparing each time point to the preceding time point within the same strain.

Table S2: Plasmids used in this study

Plasmid	Description	Antibiotic	Reference
pKMW3	Plasmid for delivery of barcoded himar transposon	Km	(1)
pNPTS138 (pDH100)	Suicide plasmid for making unmarked deletions in *Pseudomonas protegens* Pf-5; carries *sacB* for counter-selection	Km	M. R. Alley, unpublished
pGS59	To delete *lapA;* Gibson cloning of fused upstream and downstream regions of PFL_0133 into pDH100	Km	This work
pGS125	To delete *gacS;* Gibson cloning of fused upstream and downstream regions of PFL_4451 into pDH100	Km	This work
pGS124	To delete *gacA;* Gibson cloning of fused upstream and downstream regions of PFL_3563 into pDH100	Km	This work
pGS117	To delete *PFL_1588;* Gibson cloning of fused upstream and downstream regions of PFL_1588 into pDH100	Km	This work
pGS324	To delete *PFL_4192;* Gibson cloning of fused upstream and downstream regions of PFL_4192 into pDH100	Km	This work
pGS318	To delete *PFL_2828;* Gibson cloning of fused upstream and downstream regions of PFL_2828 into pDH100	Km	This work
pGS193	To delete *cheY;* Gibson cloning of fused upstream and downstream regions of PFL_1668 into pDH100	Km	This work
pGS115	To delete *PFL_0502;* Gibson cloning of fused upstream and downstream regions of PFL_0502 into pDH100	Km	This work
pGS133	To delete *PFL_5779;* Gibson cloning of fused upstream and downstream regions of PFL_5779 into pDH100	Km	This work
pGS126	To delete *PFL_4532;* Gibson cloning of fused upstream and downstream regions of PFL_4532 into pDH100	Km	This work
pGS326	To delete *PFL_1513;* Gibson cloning of fused upstream and downstream regions of PFL_1513 into pDH100	Km	This work
pGS66	To delete *fliF;* Gibson cloning of fused upstream and downstream regions of PFL_1638 into pDH100	Km	This work
pGS114	pUCT18T-miniTn7T-Gm-pBAD-AlgL used as a backbone to create construct for delivery into the Tn7 site.	Gm	(2)

pGS181	pBAD-AlgL in pGS114 omitted to insert P_{rsmX}-mVenus transcriptional reporter into the Tn7 site. RBS used in (2) positioned upstream of mVenus.	Gm	This work
pGS179	pBAD-AlgL in pGS114 omitted to insert P_{rsmY}-mVenus transcriptional reporter into the Tn7 site. RBS used in (2) positioned upstream of mVenus.	Gm	This work
pGS205	pBAD-AlgL in pGS114 omitted to insert P_{rsmZ}-mVenus transcriptional reporter into the Tn7 site. RBS used in (2) positioned upstream of mVenus.	Gm	This work
pGS159	Helper plasmid pJMP1039, carries the *tnsABCD* genes to facilitate transposition of constructs inserted into pUCT18T-miniTn7T into the Tn7 attachment site.	Carb	(3)
pGS484	To insert P_{lacUV5}-mVenus-rrnB1 cassette into CTX phage insertion site. DNA corresponding to upstream and downstream regions of CTXφ insertion site flanking an mVenus cassette were synthesized and inserted into pDH100 by GENEWIZ from Azenta Life Sciences.	Gm	This work
pGS442	*AlgL* in pGS114 omitted to generate an arabinose-inducible *gacS* expression cassette for mutant complementation.	Gm	This work
pGS328	*AlgL* in pGS114 omitted to generate an arabinose-inducible *gacA* expression cassette for mutant complementation.	Gm	This work
pGS327	*AlgL* in pGS114 omitted to generate an arabinose-inducible *fliF* expression cassette for mutant complementation.	Gm	This work
pConRef-2H12.D11	c-di-GMP fluorescent reporter. Addgene plasmid #221151.	Gm	(4)

Table S3: Strains used in this study

Strain	Organism	Genotype	Description	Source
GS94	*E. coli*	TOP10	Cloning strain	Invitrogen
GS307	*E. coli*	WM3064	Donor strain for biparental conjugation	(5)
GS11	*Pseudomonas protegens* Pf-5	Pf-5	Wild Type	ATCC BAA-477
GS14	*Agrobacterium rhizogenes*	15834	Wild Type	ATCC 15834
GS83	*P. protegens* Pf-5	Δ*lapA*	In frame deletion of *PFL_0133*	This work
GS143	*P. protegens* Pf-5	Δ*gacS*	In frame deletion of *PFL_4451*	This work
GS141	*P. protegens* Pf-5	Δ*gacA*	In frame deletion of *PFL_3563*	This work
GS137	*P. protegens* Pf-5	Δ*PFL_1588*	In frame deletion of *PFL_1588*	This work
GS345	*P. protegens* Pf-5	Δ*PFL_4192*	In frame deletion of *PFL_4192*	This work
GS335	*P. protegens* Pf-5	Δ*PFL_2828*	In frame deletion of *PFL_2828*	This work
GS343	*P. protegens* Pf-5	Δ*cheY*	In frame deletion of *PFL_1668*	This work
GS135	*P. protegens* Pf-5	Δ*PFL_0502*	In frame deletion of *PFL_0502*	This work
GS166	*P. protegens* Pf-5	Δ*PFL_5779*	In frame deletion of *PFL_5779*	This work
GS149	*P. protegens* Pf-5	Δ*PFL_4532*	In frame deletion of *PFL_4532*	This work
GS353	*P. protegens* Pf-5	Δ*PFL_1513*	In frame deletion of *PFL_1513*	This work
GS81	*P. protegens* Pf-5	Δ*fliF*	In frame deletion of *PFL_1638*	This work
GS169	*P. protegens* Pf-5	Δ*fliF*Δ*gacA*	In frame deletion of *PFL_1638*, *PFL_3563*	This work
GS455	*P. protegens* Pf-5	Wild type/CTXφ:mVenus	pGS484 integrated at CTXφ locus of GS11	This work

GS475	*P. protegens* Pf-5	Δ*gacS*/CTXφ:mVenus/Tn7:*gacS*	pGS484 integrated at CTXφ locus and pGS442 integrated at Tn7 locus of GS143	This work
GS479	*P. protegens* Pf-5	Δ*gacA*/CTXφ:mVenus/Tn7:*gacA*	pGS484 integrated at CTXφ locus and pGS328 integrated at Tn7 locus of GS141	This work
GS481	*P. protegens* Pf-5	Δ*fliF*/CTXφ:mVenus/Tn7:*fliF*	pGS484 integrated at CTXφ locus and pGS327 integrated at Tn7 locus of GS81	This work
GS235	*P. protegens* Pf-5	Wild type/Tn7:P_{rsmX}-mVenus	pGS181 integrated at Tn7 locus of GS11	This work
GS237	*P. protegens* Pf-5	Wild type/Tn7:P_{rsmY}-mVenus	pGS179 integrated at Tn7 locus of GS11	This work
GS239	*P. protegens* Pf-5	Wild type/Tn7:P_{rsmZ}-mVenus	pGS205 integrated at Tn7 locus of GS11	This work
GS242	*P. protegens* Pf-5	Δ*gacA*/Tn7:P_{rsmX}-mVenus	pGS181 integrated at Tn7 locus of GS141	This work
GS244	*P. protegens* Pf-5	Δ*gacA*/Tn7:P_{rsmY}-mVenus	pGS179 integrated at Tn7 locus of GS141	This work
GS246	*P. protegens* Pf-5	Δ*gacA*/Tn7:P_{rsmZ}-mVenus	pGS205 integrated at Tn7 locus of GS141	This work
GS249	*P. protegens* Pf-5	Δ*fliF*/Tn7:P_{rsmX}-mVenus	pGS181 integrated at Tn7 locus of GS81	This work
GS251	*P. protegens* Pf-5	Δ*fliF*/Tn7:P_{rsmY}-mVenus	pGS179 integrated at Tn7 locus of GS81	This work

GS253	*P. protegens* Pf-5	Δ*fliF*/Tn7:P_{rsmZ}-mVenus	pGS205 integrated at Tn7 locus of GS81	This work

Table S4: Primers used in this study

Primer ID	Name	Sequence (5' to 3')	Purpose
1	d_0133a	GCTACGTAATACGACTCACTAGTGGGTTCAGATCAATCCGGTTTCATC	Cloning of fused up- and down-stream flanking regions of *PFL_0133* into pDH100
2	d_0133b	ATGAGCAGTGTTAACAACAGCTGACCGGTCC	
3	d_0133c	TCAGCTGTTGTTAACACTGCTCATTGGGGAC	
4	d_0133d	CCAGATATCCTGCAGAGAAGCTTGGCTTTCAGCTGGTAGTCAG	
5	d_4451a	GCTACGTAATACGACTCACTAGTGAATGGCTTGGGCAGGTAATC	Cloning of fused up- and down-stream flanking regions of *PFL_4451* into pDH100
6	d_4451b	TCAGGCGTTGGTTTTCTTGAGCACGCAACTCTC	
7	d_4451c	GTGCTCAAGAAAACCAACGCCTGACCCAGG	
8	d_4451d	CCAGATATCCTGCAGAGAAGCTTCACGGTCTTGCCCACCAG	
9	d_3563a	GCTACGTAATACGACTCACTAGTGATCTGGTACTGCAGGCAG	Cloning of fused up- and down-stream flanking regions of *PFL_3563* into pDH100
10	d_3563b	TTGATAAGGGTGGCCAGCCTCTGAAATGACC	
11	d_3563c	TCAGAGGCTGGCCACCCTTATCAAGCAGACAC	
12	d_3563d	CCAGATATCCTGCAGAGAAGCTTCATCCAAATGAGTGGAATCGG	
13	d_1588a	GCTACGTAATACGACTCACTAGTTAGAAGGGCACCTTGCCG	Cloning of fused up- and down-stream flanking regions of *PFL_1588* into pDH100
14	d_1588b	TCACGGTGTTCGTCGTTCGCTCATGAGGGG	
15	d_1588c	ATGAGCGAACGACGAACACCGTGATGATTTCC	
16	d_1588d	CCAGATATCCTGCAGAGAAGCTTGTACTGGTGGACCACCAAC	
17	d_4192a	GCTACGTAATACGACTCACTAGTGGTCAACTTCATGATTCGCGATG	Cloning of fused up- and down-stream flanking regions of *PFL_4192* into pDH100
18	d_4192b	ATGTCCTTGCCGCCCCACGCCTGACATCAC	
19	d_4192c	TCAGGCGTGGGGCGGCAAGGACATCAAGGCAAC	
20	d_4192d	CCAGATATCCTGCAGAGAAGCTTCAGCATCTTTCATCACTTCAAGAGC	
21	d_2828a	GCTACGTAATACGACTCACTAGTACTATCTGCAGTCGGTCAG	Cloning of fused up- and down-stream flanking regions of *PFL_2828* into pDH100
22	d_2828b	TCAGACGAACCAGAGAAATGACATCGGTGACTC	
23	d_2828c	ATGTCATTTCTCTGGTTCGTCTGAACGGAC	
24	d_2828d	CCAGATATCCTGCAGAGAAGCTTCTGGATGACGAGGGGATTG	
25	d_1668a	GCTACGTAATACGACTCACTAGTCAGTTTCGACGACCTGTTG	Cloning of fused up- and down-stream

26	d_1668b	TCAACCGATGCGGAGGATTTTCATGTTCTTGTCC	flanking regions of *PFL_1668* into pDH100
27	d_1668c	ATGAAAATCCTCCGCATCGGTTGAGACGCG	
28	d_1668d	CCAGATATCCTGCAGAGAAGCTTCGTTGAGGTGGCCGGAAAC	
29	d_0502a	GCTACGTAATACGACTCACTAGTAGCAGGCGTACGTTGTCTAC	Cloning of fused up- and down-stream flanking regions of *PFL_0502* into pDH100
30	d_0502b	ATGCGCCCCAACCCACAGCCCTGAACCAGG	
31	d_0502c	TCAGGGCTGTGGGTTGGGGCGCATGGTGTTTTC	
32	d_0502d	CCAGATATCCTGCAGAGAAGCTTAGGTAGGGAATCGCCACCATG	
33	d_5779a	GCTACGTAATACGACTCACTAGTGAACTCCAGCTGGTGATAG	Cloning of fused up- and down-stream flanking regions of *PFL_5779* into pDH100
34	d_5779b	ATGGAATGCGTGCTACAGGCCTGAATTCGC	
35	d_5779c	TCAGGCCTGTAGCACGCATTCCATGCTGCTTC	
36	d_5779d	CCAGATATCCTGCAGAGAAGCTTGTTTTCGAGCCGGTCACCG	
37	d_4532a	GCTACGTAATACGACTCACTAGTCTGTTCGCCGAGATCCTG	Cloning of fused up- and down-stream flanking regions of *PFL_4532* into pDH100
38	d_4532b	TTATGCCATTTGTTGGTTTTCCATAAATTCCTGC	
39	d_4532c	ATGGAAAACCAACAAATGGCATAAAAACCCAAAAAAC	
40	d_4532d	CCAGATATCCTGCAGAGAAGCTTGCCTGTCACCTGGTCTTC	
41	d_1513a	GCTACGTAATACGACTCACTAGTGCCATGGTGATCTTCGGCTCTTAC	Cloning of fused up- and down-stream flanking regions of *PFL_1513* into pDH100
42	d_1513b	TCATAGGCCGGACAGGCGGCTCATGGTCAGTG	
43	d_1513c	ATGAGCCGCCTGTCCGGCCTATGAAGCCACAG	
44	d_1513d	CCAGATATCCTGCAGAGAAGCTTCGCTCGCGGTCGAACAAC	
45	d_1638a	GCTACGTAATACGACTCACTAGTGACCTTGCGCTACAAGTTG	Cloning of fused up- and down-stream flanking regions of *PFL_1638* into pDH100
46	d_1638b	TCACTCATCTGCTGCTTCTGCCATGACTCAATC	
47	d_1638c	ATGGCAGAAGCAGCAGATGAGTGATAACCGAGC	
48	d_1638d	CCAGATATCCTGCAGAGAAGCTTTCGAGAATCTGGTTGAGTTCC	
49	mVenus_F_NcoI	AAATTTCCATGGAGGAGGATATTCATGGTGAGCAAGGGCGAGG	Cloning of RBS-mVenus-rrnB1 into pGS114 to generate pGS181, pGS179, and pGS205
50	mVenus_R_SacI	AAATTTGAGCTCCGGCGCGCCATTGGGATG	
51	RsmX_F_HindIII	AAATTTAAGCTTACTAGGGGTTTGCGTAAGTG	

52	RsmX_R_NcoI	AAATTTCCATGGGAAGATTAAACACAAAGCCCGG	Cloning of P_{rsmX} into pGS114 to generate pGS181
53	RsmY_F_HindIII	AAATTTAAGCTTCCAGCAAGGGCGTTTGTAAG	Cloning of P_{rsmY} into pGS114 to generate pGS179
54	RsmY_R_NcoI	AAATTTCCATGGGTGAAGTAGATTAGCTTCAGCCG	
55	RsmZ_F_HindIII	AAATTTAAGCTTCCACTGGATCGATTCACAAG	Cloning of P_{rsmZ} into pGS114 to generate pGS205
56	RsmZ_R_NcoI	AAATTTCCATGGCAGGGCTGATATTAGAGAGTTC	
57	AlgL_cut_F	AGGAGGATATTCGAGCTCATGCATGATCGAATTAG	Gibson excision of AlgL from pGS114
58	AlgL_cut_R	ATGCATGAGCTCGAATATCCTCCTCCCATGGC	
59	gacS_F_NotI	AAATTTGCGGCCGCGAGGAGGATATTCGTGCTCAAGAAACTGGGAATCAAAGGC	Cloning of *gacS* into pGS114 following excision of AlgL using primers 57 and 58 to generate pGS442
60	gacS_R_SacI	AAATTTGAGCTCTCAGGCGTTGGTCCGCGC	
61	gacA_F_XmaI	AAATTTCCCGGGGAGGAGGATATTCTTGATAAGGGTGCTAGTAGTCGATG	Cloning of *gacA* into pGS114 to generate pGS328
62	gacA_R_SacI	AAATTTGAGCTCTCAGAGGCTGGCATCAACC	
63	fliF_F_XmaIGib	CTCCATACCCGTTTTTTTGCGAGGAGGATATTCATGGCAGAAGCAGTCGCC	Cloning of *fliF* into pGS114 to generate pGS327
64	fliF_R_SacIGib	AATTCGATCATGCATGAGCTTCACTCATCTGCGTTAATCCACTC	

References:

1. Wetmore KM, Price MN, Waters RJ, Lamson JS, He J, Hoover CA, Blow MJ, Bristow J, Butland G, Arkin AP, Deutschbauer A. 2015. Rapid Quantification of Mutant Fitness in Diverse Bacteria by Sequencing Randomly Bar-Coded Transposons. mBio 6:e00306-15–15.

2. Gheorghita AA, Wolfram F, Whitfield GB, Jacobs HM, Pfoh R, Wong SSY, Guitor AK, Goodyear MC, Berezuk AM, Khursigara CM, Parsek MR, Howell PL. 2022. The Pseudomonas aeruginosa homeostasis enzyme AlgL clears the periplasmic space of accumulated alginate during polymer biosynthesis. Journal of Biological Chemistry 298.

3. Peters JM, Koo B-M, Patino R, Heussler GE, Hearne CC, Qu J, Inclan YF, Hawkins JS, Lu CHS, Silvis MR, Harden MM, Osadnik H, Peters JE, Engel JN, Dutton RJ, Grossman AD, Gross CA, Rosenberg OS. 2019. Enabling genetic analysis of diverse bacteria with Mobile-CRISPRi. Nat Microbiol 4:244–250.

4. Kaczmarczyk A, Van Vliet S, Jakob RP, Teixeira RD, Scheidat I, Reinders A, Klotz A, Maier T, Jenal U. 2024. A genetically encoded biosensor to monitor dynamic changes of c-di-GMP with high temporal resolution. Nat Commun 15:3920.

5. Dehio C, Meyer M. 1997. Maintenance of broad-host-range incompatibility group P and group Q plasmids and transposition of Tn5 in Bartonella henselae following conjugal plasmid transfer from Escherichia coli. J Bacteriol 179:538–540.
